# Prevalence and risk of psychological distress, anxiety and depression in adolescent and young adult (AYA) cancer survivors: A systematic review and meta‐analysis

**DOI:** 10.1002/cam4.6435

**Published:** 2023-08-10

**Authors:** Vanesa Osmani, Lucy Hörner, Stefanie J. Klug, Luana Fiengo Tanaka

**Affiliations:** ^1^ Chair of Epidemiology, TUM Department of Sport and Health Sciences Technical University of Munich Munich Germany

**Keywords:** anxiety, AYA cancer survivors, depression, psychological distress, systematic review

## Abstract

**Background:**

Adolescent and young adult (AYA) cancer survivors (CS) face unique psychosocial challenges, which may affect their mental health. However, there are inconsistencies in AYA definitions and varying prevalence data on psychological distress, anxiety, and depression. We aimed to synthesize published literature on prevalence, risk, longitudinal changes, and predictors for these outcomes and estimate pooled prevalences.

**Methods:**

We searched for observational studies published in English before June 1 2022, in PubMed, PsycINFO, Scopus, and Web of Science. Two researchers extracted independently information on study characteristics, prevalence, and risk. The pooled prevalence (PP) of psychological distress, anxiety, and depression was estimated using random‐effects models. Geographical region, treatment status, and assessment instruments were considered in stratified meta‐analyses.

**Results:**

Sixty‐eight studies were included in the systematic review and 57 in the meta‐analyses. We estimated an overall prevalence of 32% (*n* = 30; 4226/15,213 AYAs; 95% CI, 23%–42%; *I* ^2^ = 99%) for psychological distress, 29% for anxiety (*n* = 24; 2828/8751 AYAs; 95% CI, 23%–36%; *I* ^2^ = 98%), and 24% (*n* = 35; 3428/16,638 AYAs; 95% CI, 18%–31%; *I* ^2^ = 98%) for depression. The range of PP of psychological distress varied across geographical regions, treatment status, and assessment instruments. The PP of anxiety varied significantly across continents, while no variations were seen for depression. Studies found higher risks for psychological distress, anxiety, and depression in AYAs compared to older cancer survivors or cancer‐free peers.

**Conclusions:**

Our research found that one in three AYA‐CS experience psychological distress or anxiety and one in four are affected by depression, highlighting the need for specialized psychological services for AYA‐CS in oncology settings and AYA‐focused interventions.

## 1 INTRODUCTION

Adolescent and young adult (AYA) cancer survivors (CS) are defined by the US National Cancer Institute (NCI) as those diagnosed between 15 and 39 years.^1^ In 2020, there were 1,233,225 incident and 3,230,897 prevalent cancers among AYA‐CS worldwide.^2^ Cancer survivorship, starting at cancer diagnosis, can be a demanding time for these individuals. Along with the cancer experience, AYA‐CS often face challenges related to sexual health, fertility, relationship formation, education, and work.^3^, ^4^ Studies have shown that AYA‐CSs' needs including those for information, connection with others and financial support remain often unmet.^5^, ^6^

Previous research found that AYA‐CS are more psychologically distressed than other CS and have a higher risk of psychiatric disorders in comparison with healthy peers; however, these data were based on few studies and disregarded longitudinal changes.^7^, ^8^, ^9^ Other reviews have not distinguished between AYA survivors of childhood cancers (diagnosed before 15 years) and AYA‐onset CS,^10^ or focused on specific subgroups of AYA‐CS, overlooking young adult‐CS.^11^, ^12^, ^13^ None of these reviews estimated the prevalence of psychological outcomes in this population nor did they take into account potential geographical differences in prevalence rates. Given that high levels of distress may impact coping with cancer, health‐related quality of life and survival,^14^ comprehensive epidemiological data on these outcomes are needed to guide future prevention efforts for AYA‐CS. Additionally, investigating geographical variations can help in identifying disparities in research and care, which could inform the development of targeted interventions and policy recommendations.

We aimed to systematically review and summarize the literature on prevalence, risk and associated factors, and trajectories for psychological distress, anxiety, and depression among AYA‐CS, and estimate the prevalence of these outcomes in meta‐analyses.

## 2 METHODS

The protocol of this systematic review was registered in the International Prospective Register of Systematic Reviews (PROSPERO, ID: CRD42020175991). This work was performed following the Preferred Reporting Items in Systematic Reviews and Meta‐analyses (PRISMA) and Meta‐analyses Of Observational Studies in Epidemiology (MOOSE) statements.

### 2.1 Eligibility criteria

Study eligibility criteria were defined using the PECOS (population, exposure, comparison, outcome and study design) scheme (eTable 1). The population of interest were AYA‐CS (15–39 years at any cancer diagnosis).^1^ To account for various existing definitions of AYA‐CS, we considered studies including participants as AYAs with a lower or upper age range (+/−5 years from the NCI definition). When age at diagnosis was not reported, the time since diagnosis and the current participant's age was considered. The comparison groups included cancer‐free peers, older and younger CS, and siblings.

The outcomes of interest were as follows: (1) psychological distress (also as an overall measure of both anxiety and depression), (2) anxiety, and (3) depression (also reported as mood disorders). The studies were included if the outcomes were assessed via screeners using clinical cutoffs, clinical interviews, and diagnoses based on the International Classification of Diseases (ICD‐10) reporting or Diagnostic and Statistical Manual of Mental Disorders 4th or 5th Edition (DSM‐4 or 5) criteria for mental disorders, or self‐disclosed diagnoses. We extracted prevalence, risk ratios (RR), hazard ratios (HR), odds ratios (OR), or *p*‐values for prevalence and mean comparisons between groups and over time. Observational studies (cross‐sectional, cohort or case–control) published in English were included if they focused on AYA‐CS or if they reported stratified results for this population. Other study types were excluded.

### 2.2 Search strategy and eligibility assessment

We systematically searched four databases (Medline via PubMed, PsycINFO, Scopus, and Web of Science) from inception to May 31, 2022 (eTable 2). Additionally, hand searching was conducted using simple search terms in Google Scholar and screening the reference lists of prior reviews. The results of the search were managed with Endnote X9. Authors were contacted to provide the full‐texts if these were not accessible. Two authors (VO and LH) independently screened the studies for eligibility, with discrepancies resolved through discussion and, if needed, consultation with a third reviewer (LFT).

### 2.3 Data extraction and quality assessment

We extracted data on general study information (authors, country, study design, and duration), participant information (age at diagnosis, current age, sex, cancer type, recruitment sources of CS and controls, if applicable, and treatment status), outcome definition, assessment time, instruments used, prevalence estimates, risk measures, and findings on predictors (if reported). Two authors (VO and LH) extracted the data independently using Microsoft Excel. Results were compared and any discrepancies were discussed.

We used a modified Newcastle‐Ottawa quality assessment scale (NOS) to assess study quality.^15^ NOS evaluates the representativeness of the sample, sample size, non‐respondents, exposure ascertainment, comparability of subjects in different outcome groups, outcome assessment, and statistical methods (eFigure 1 and 2). Studies were rated based on a star system, with a maximum score of 10. Very good studies were considered those receiving 9–10 stars, good studies with 8–9 stars, satisfactory with 5–6 stars, and unsatisfactory those with 0–4 stars.

### 2.4 Data synthesis and analysis

We report study characteristics and quality, prevalence, and longitudinal changes by outcome and summarize the evidence on frequently reported predictors from individual studies. We present forest plots of OR and RR from individual studies by comparison group (cancer‐free peers, older and younger CS or siblings). We did not pool these estimates due to a limited number of studies for each outcome.

Mean pooled prevalence (PP) of psychological distress, anxiety, and depression was calculated using random‐effects models in meta‐analyses with the restricted maximum likelihood (REML) as an estimator. Only studies reporting on prevalence where population numbers were available were included in these analyses. For longitudinal studies, only the prevalence at baseline was included, considering possible dropouts in the follow‐up. We additionally conducted sensitivity analyses by excluding studies with unsatisfactory quality assessment.

PP and 95% confidence intervals (95% CIs) are presented in forest plots separately for each outcome. Between‐study heterogeneity was examined using *I* ^2^, Cochran's *Q* statistic, and ${Χ}^{2}$ tests. To explore reasons for heterogeneity, stratified meta‐analyses were conducted for each outcome where ${Χ}^{2}$ tests were performed to test for any differences between the subgroups. Treatment status, continent, assessment instruments, and corresponding cutoffs were considered in these analyses. We assessed publication bias using Egger's test. Statistical analyses were two‐sided with a significance level of 5%. They were conducted in R version 4.1.2 using the packages “meta” and “metafor.”

## 3 RESULTS

**FIGURE 1:**
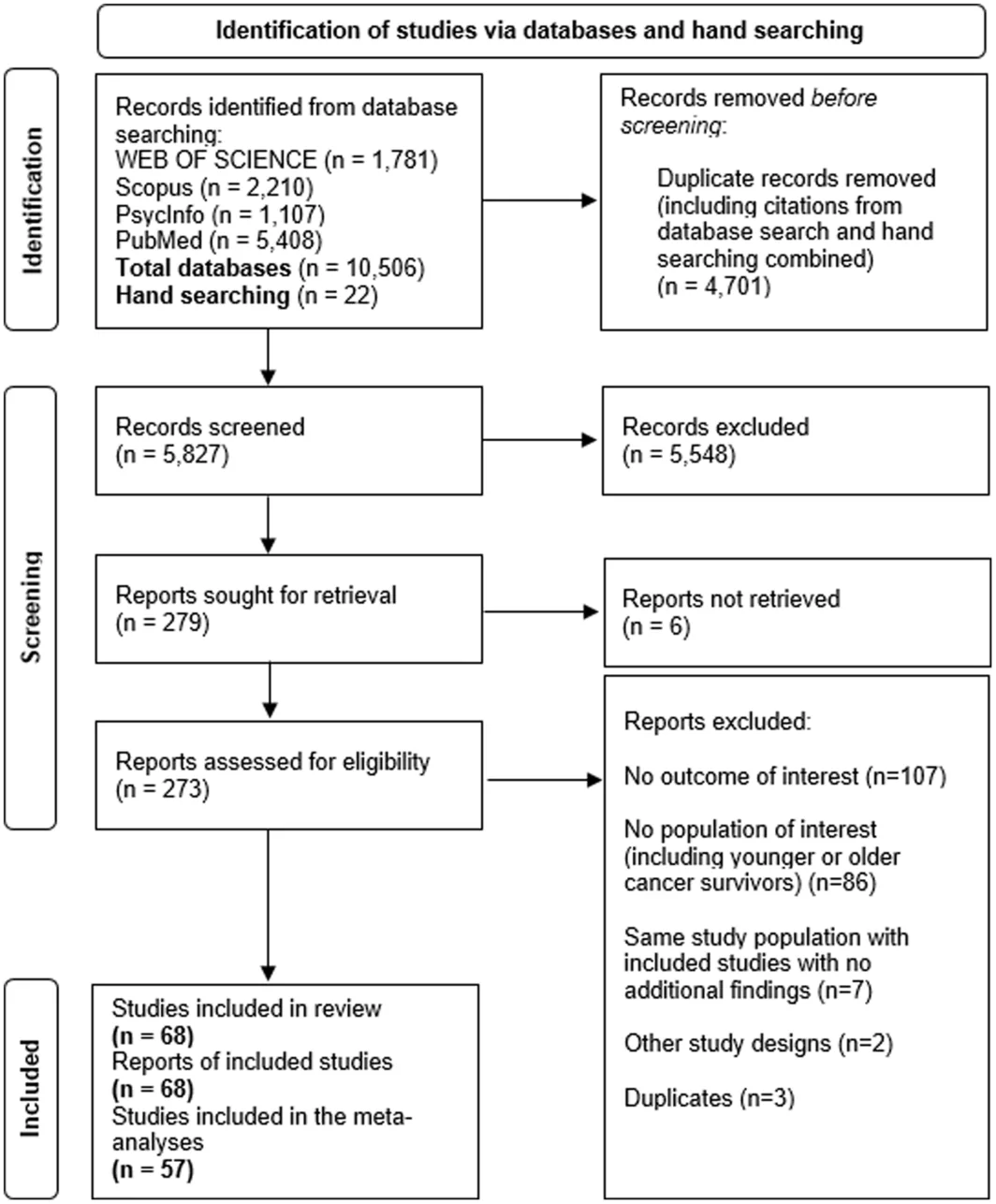
Modified PRISMA flowchart illustrating the study selection process.

Overall, 10,506 records were identified from databases and 22 studies via hand searching (Figure 1). After removing duplicates, 5827 records were screened for title and abstract and 279 reports were considered for further screening; 6 studies could not be retrieved.^16^, ^17^, ^18^, ^19^, ^20^, ^21^ After screening 273 full‐text articles, 68 studies met the eligibility criteria and were included in the qualitative synthesis and 57 studies which reported prevalence and had information on population numbers were included in the meta‐analyses.

### 3.1 Study characteristics

The study characteristics are summarized in Table 1 and eTable 3. The majority of the studies were conducted in North America (45.6%)^22^, ^23^, ^24^, ^25^, ^26^, ^27^, ^28^, ^29^, ^30^, ^31^, ^32^, ^33^, ^34^, ^35^, ^36^, ^37^, ^38^, ^39^, ^40^, ^41^, ^42^, ^43^, ^44^, ^45^, ^46^, ^47^, ^48^, ^49^, ^50^, ^51^, ^52^ and Europe (26.5%).^53^, ^54^, ^55^, ^56^, ^57^, ^58^, ^59^, ^60^, ^61^, ^62^, ^63^, ^64^, ^65^, ^66^, ^67^, ^68^, ^69^, ^70^ Fifty‐two studies^22^, ^23^, ^24^, ^25^, ^27^, ^30^, ^31^, ^32^, ^33^, ^34^, ^35^, ^36^, ^38^, ^39^, ^40^, ^41^, ^42^, ^43^, ^44^, ^46^, ^47^, ^48^, ^50^, ^51^, ^54^, ^56^, ^57^, ^58^, ^59^, ^62^, ^63^, ^65^, ^67^, ^68^, ^69^, ^70^, ^71^, ^72^, ^73^, ^74^, ^75^, ^76^, ^77^, ^78^, ^79^, ^80^, ^81^, ^82^, ^83^, ^84^, ^85^, ^86^ were cross‐sectional and 16 longitudinal.^26^, ^28^, ^29^, ^37^, ^45^, ^49^, ^52^, ^53^, ^55^, ^60^, ^61^, ^64^, ^66^, ^87^, ^88^, ^89^ More than half recruited participants from a clinical setting (e.g., hospitals), 11 studies (16.1%) through cancer registries and only nine (13.2%) from the general population.^22^, ^25^, ^33^, ^36^, ^39^, ^43^, ^46^, ^83^, ^86^ The majority of the studies (72%) had less than 500 participants, and 66.2% included any cancer type. As for treatment status, 38.2% of the studies recruited participants who were off treatment at the assessment time.^24^, ^25^, ^27^, ^30^, ^32^, ^33^, ^34^, ^35^, ^36^, ^39^, ^42^, ^43^, ^44^, ^46^, ^47^, ^50^, ^53^, ^57^, ^60^, ^61^, ^65^, ^67^, ^68^, ^70^, ^79^, ^85^

**TABLE 1:** Summary of the main characteristics of the included studies (*n* = 68).

Study characteristics	Number (%)
World region	
North America	31 (45.6)
Europe	18 (26.5)
Asia	11 (16.1)
Oceania	7 (10.3)
Multiple regions	1 (1.5)
Study design	
Cross‐sectional	52 (76.5)
Longitudinal	16 (23.5)
Recruitment setting	
Clinical setting (clinics, cancer centers, hospitals, clinical databases)	36 (52.9)
Cancer registries	11 (16.1)
Multiple sources	10 (14.7)
General population	9 (13.2)
Other	2 (2.9)
Number of AYA cancer survivors	
<100	22 (32.4)
100–300	18 (26.5)
301–500	9 (13.2)
500+	19 (28.0)
Age at diagnosis	
Adolescence and young adulthood (range 12–40 years)	47 (86.8)
Young adulthood (range 18–45)	16 (23.5)
Adolescence (range 13–19)	5 (7.4)
Type of cancer	
Any cancer type^a^	45 (66.2)
Breast and gynecological	9 (13.2)
Primarily hematological	8 (11.8)
Other	6 (8.8)
Treatment status	
Off treatment	26 (38.2)
On treatment	18 (26.5)
Majority off treatment	11 (16.2)
Majority on treatment	13 (19.1)
Reported outcomes of interest	
All three outcomes	10 (14.7)
Anxiety and depression	26 (38.2)
Only psychological distress	21 (30.8)
Only depression	10 (14.7)
Only anxiety	1 (1.5)
Instruments used to assess outcome^b^	
Psychological distress (*n* = 31)	
K6 and K10	9 (29.0)
DT	8 (25.8)
BSI‐18	5 (16.1)
HADS	3 (9.7)
Clinical diagnoses	2 (6.5)
Other	4 (12.9)
Anxiety (*n* = 37)	
HADS	14 (37.8)
STAI	4 (10.8)
GAD‐7	3 (8.1)
PSSCAN‐R	3 (8.1)
BSI‐18	3 (8.1)
Multiple instruments including clinical diagnoses	3 (8.1)
Other	7 (18.9)
*Depression (n = 46)*	
HADS	12 (26.1)
CES‐D	9 (19.6)
PHQ‐8 and 9	5 (10.9)
Clinical diagnoses^c^	6 (13.0)
PSSCAN‐R	3 (6.5)
BSI‐18	3 (6.5)
Other	8 (17.4)
Study quality based on NOS	
Very good	4 (5.9)
Good	17 (25.9)
Satisfactory	32 (47.0)
Unsatisfactory	15 (22.1)

Note: Percentages may not add up to 100% due to rounding;

Abbreviations: AYA, adolescent and young adults; K6, The Kessler Psychological Distress Scale – 6; K10, The Kessler Psychological Distress Scale – 10; DT, Distress Thermometer; BSI ‐18, Brief Symptom Inventory – 18; HADS, The Hospital Anxiety and Depression Scale; STAI, State–Trait Anxiety Inventory; GAD‐7, Generalized Anxiety Disorder Scale −7; PSSCAN‐R, The Psychosocial Screen for Cancer; CES‐D, Center for Epidemiologic Studies Depression Scale; PHQ‐8, Patient Health Questionnaire‐8; PHQ‐9, Patient Health Questionnaire‐9; NOS, Newcastle‐Ottawa Scale.

^a^ Including all cancers without any exclusion or all common cancers.

^b^ Including all studies reporting on prevalence, risk estimates, and/or comparisons between groups and across time points. Not all these studies were included in the meta‐analysis because they did not report on prevalence or AYA participant numbers.

^c^ It also includes studies, which identified the population from cancer registries and the psychological diagnoses from hospital registries.

Regarding study quality, only four (5.9%)^53^, ^59^, ^64^, ^88^ were considered of very good and 17 (25.9%)^22^, ^25^, ^33^, ^38^, ^42^, ^43^, ^44^, ^50^, ^52^, ^57^, ^62^, ^66^, ^68^, ^71^, ^72^, ^81^, ^90^ of good quality. The rest of the studies were rated as satisfactory (47%) or unsatisfactory (22.1%) (Table 1 and eTable 4).

### 3.2 Psychological distress prevalence

**FIGURE 2:**
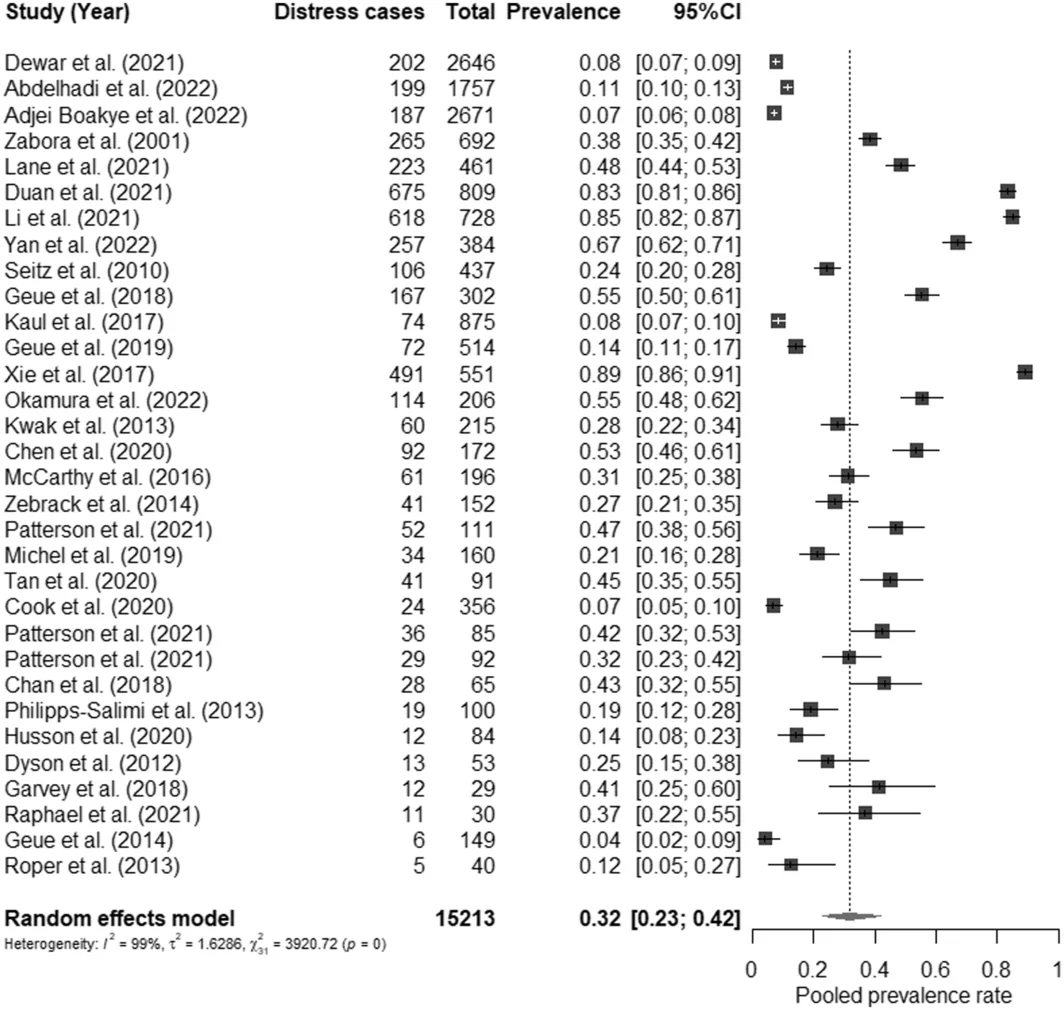
Meta‐analysis results on prevalence of psychological distress among AYA cancer survivors (30 studies; 15,213 participants). Patterson and colleagues (2021) reported on three different world regions and the respective prevalences have been included separately. The vertical dashed line indicates the crude pooled prevalence.

Thirty studies reported psychological distress prevalence, with estimates ranging from 4% to 89% (Figure 2, eTable 5). The PP of psychological distress based on 15,213 AYA‐CS was 32% (95% CI, 23%–42%) (Figure 2). The between‐study heterogeneity was significant (*I* ^*2*^ = 99%, *p* = 0). Similar results were seen after excluding studies with unsatisfactory quality assessment (eFigure 18–21).^51^, ^83^, ^87^, ^89^

The highest prevalence rates of psychological distress were reported in Asia (PP 71%; 95% CI 52%–84%) followed by Oceania (PP 36%; 95% CI, 18%–58%), while the lowest in North America (PP 22%; 95% CI, 14%–32%) and Europe (PP 22%; 95% CI, 11%–37%) [*χ* ^2^ test, *p* < 0.01] (eFigure 3). Studies including participants undergoing cancer treatment had the highest prevalence (PP 62%; 95% CI, 37%–82%), followed by those including mainly on‐treatment CS (PP 38%; 95% CI, 24%–55%). The lowest prevalence of psychological distress was among AYA‐CS, where a majority (PP 26%; 95% CI, 12%–49%) or all had completed the cancer treatments (PP 18%; 95% CI, 10%–30%) [*χ* ^2^ test, *p* < 0.01] (eFigure 4).

The most frequently used instruments were the Kessler psychological distress scales 6 (K6) and 10 (K10), and Distress Thermometer (DT). Studies using the DT ≥4 reported the highest prevalences (PP 65%; 95% CI 49%–79%), while those using the Kessler (K6 ≥ 13) the lowest (PP 10%; 95% CI, 5%–19%) [*χ* ^2^ test, *p* < 0.01] (eFigure 5). No significant funnel plot asymmetry was detected (*z* = −1.4586, *p* = 0.1447).

### 3.3 Anxiety prevalence

**FIGURE 3:**
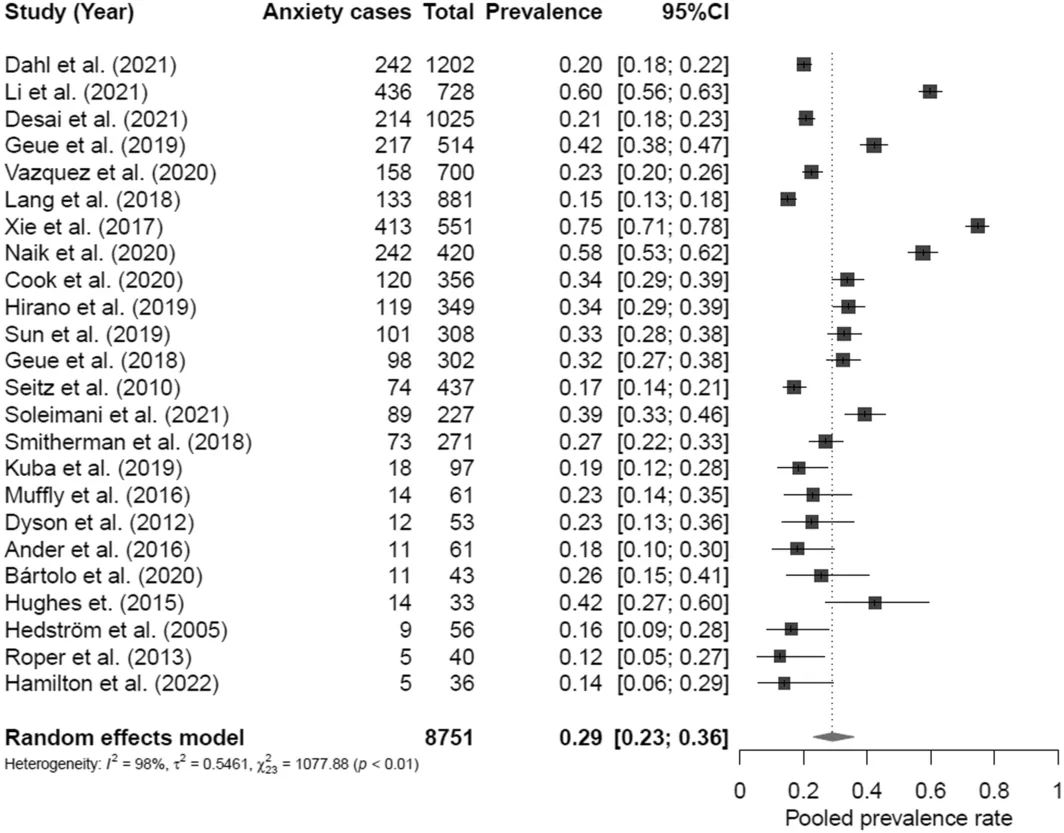
Meta‐analysis results on prevalence of anxiety among AYA cancer survivors (24 studies; 8751 participants). The vertical dashed line indicates the crude pooled prevalence.

Twenty‐four studies reported the prevalence of anxiety, with estimates ranging from 12% to 75% (Figure 3, eTable 5). The most used instrument was the Hospital Anxiety and Depression Scale (HADS). Based on these studies including 8751 participants, the PP of anxiety was 29% (95% CI, 23%–36%) (Figure 3) and a between‐study heterogeneity was detected (*I* ^*2*^ = 98%, *p* < 0.01). Studies from Asia had the highest prevalence of anxiety (PP 51%; 95% CI 36%–66%), followed by Oceania (PP 32%; 95% CI, 15%–56%) (eFigure 6). The lowest prevalences were reported in North America (PP 26%; 95% CI, 19%–35%) and Europe (PP 23%; 95% CI, 16%–33%) [*χ* ^2^ test, *p* < 0.01]. No significant differences were found based on the treatment status of AYA‐CS (eFigure 7). Similarly, no significant differences in prevalence rates were seen based on the instrument used [*χ* ^2^ test, *p* = 0.37] (eFigure 8). The test for forest plot asymmetry indicated a possible publication bias (*z* = −2.3023, *p* = 0.0213). Comparable results were seen when studies with unsatisfactory quality assessment were excluded (eFigure 22–25).^35^, ^41^, ^48^, ^49^, ^55^, ^74^

### 3.4 Depression prevalence

**FIGURE 4:**
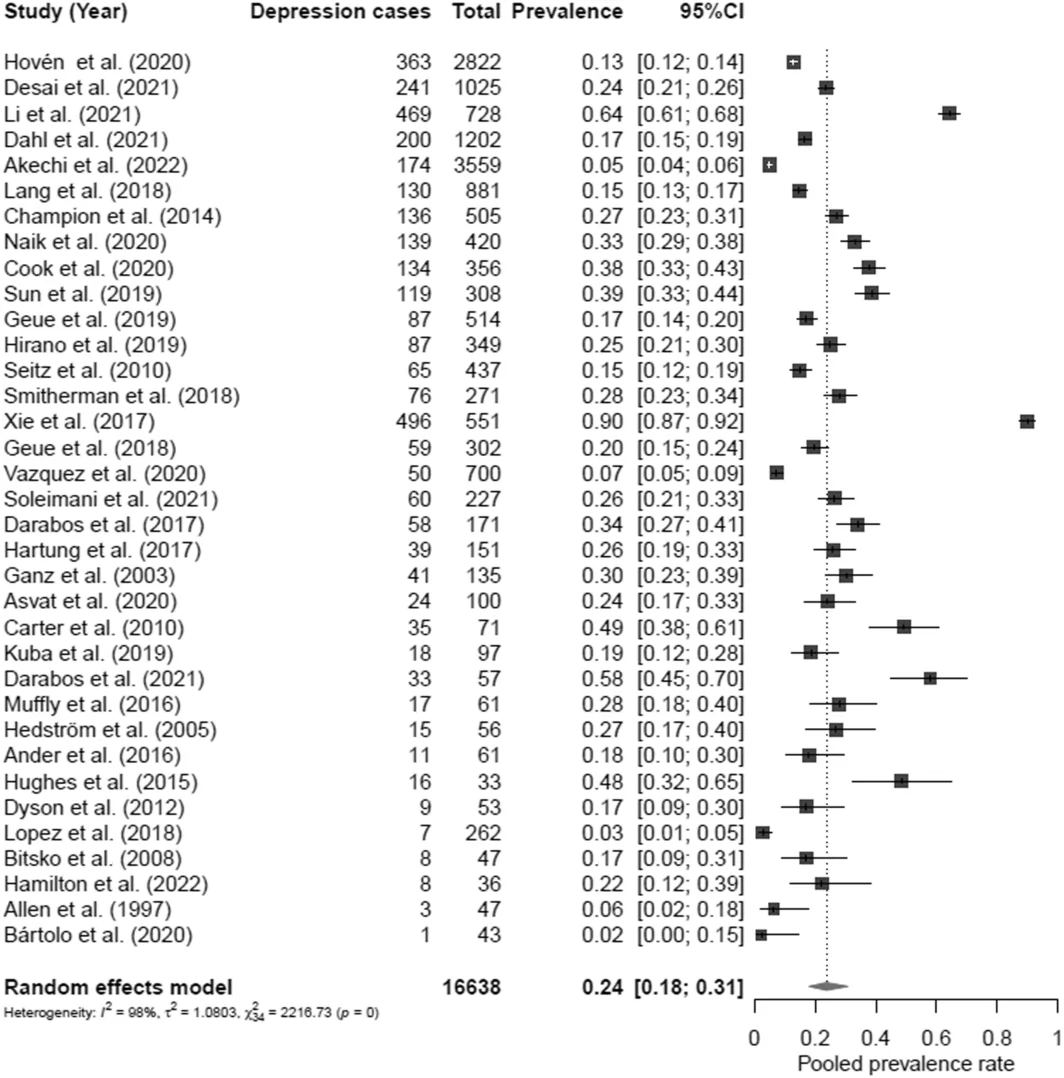
Meta‐analysis results on prevalence of depression among AYA cancer survivors (35 studies; 16,638 participants). The vertical dashed line indicates the crude pooled prevalence.

Thirty‐five studies reported on the prevalence of depression, with prevalence ranging from 2% to 90%. The most used instrument was the HADS. The summary prevalence of depression among 16,638 AYA‐CS from these studies was 24% (95% CI 18%–31%) (Figure 4). The test for between‐study heterogeneity was significant (*I* ^*2*^ = 98%, *p* = 0). No differences were detected based on the study region [*χ* ^2^ test, *p* = 0.10], treatment status [*χ* ^2^ test, *p* = 0.61] (eFigure 10), and the instrument used [*χ* ^2^ test, *p* = 0.68] (eFigure 11). The test for forest plot asymmetry indicated a possible publication bias (*z* = −2.0071, *p* = 0.0447). Excluding studies with unsatisfactory quality assessment yielded analogous findings (eFigure 26–29)^24^, ^26^, ^35^, ^41^, ^48^, ^49^, ^54^, ^55^, ^74^; however, no publication bias was detected (*z* = −1.9239, *p* = 0.0544).

### 3.5 Risk of developing psychological distress, anxiety, and depression

In comparison with older cancer survivors, AYA‐CS had approximately fourfold higher odds of developing psychological distress (eTable 6, eFigure 12).^86^ In comparison with cancer‐free peers, the odds of experiencing psychological distress were at least 1.6‐fold higher among AYA‐CS.^22^, ^33^, ^43^ The only study reporting RRs found no differences in the distress risk between AYA‐CS and older survivors or cancer‐free controls (eFigure 13).^25^

Three of four studies comparing AYA‐CS with older cancer survivors reported elevated odds of anxiety (at least 1.6 fold) among AYA‐CS (eTable 6, eFigure 14).^39^, ^42^, ^70^ Similarly, the odds of anxiety were higher among AYAs in comparison with cancer‐free peers^39^ and siblings.^44^ Analogous findings were seen in studies, reporting RRs,^53^, ^67^ however with a borderline higher risk for AYA‐CS in comparison with siblings (eFigure 15).^53^

Dahl and colleagues found no differences in depression odds between AYA‐CS and younger CS (<15 years) (eTable 6, eFigure 16).^57^ All but one study reported higher odds and risk of depression among AYA‐CS compared to older CS or cancer‐free peers (at least 1.3‐fold) (eFigures 16, 17).^39^, ^42^, ^64^, ^67^, ^70^, ^88^ Two out of three studies comparing AYA‐CS with siblings found differences in the risk of depression (eFigures 16, 17).^44^, ^53^

### 3.6 Psychological outcomes trajectories

Three out of seven studies, which investigated longitudinal changes in psychological distress identified decreases in distress levels up to 12 months since diagnosis or completion of treatment (eTable 7).^45^, ^87^, ^89^ The remaining four studies report small or no differences in distress levels up to 24 months since diagnosis.^28^, ^37^, ^52^, ^60^

Three out of five studies, assessing anxiety longitudinally among AYA‐CS, found no differences from diagnosis to after 12 months.^37^, ^45^, ^60^ In contrast, Ander and colleagues reported a decrease at 4 years from diagnosis and a higher prevalence of anxiety 10 years from cancer diagnoses.^55^ Jörngården and colleagues reported decreases in anxiety among AYA‐CS at 18 months after diagnosis.^66^

Five out of six studies assessing depression rates among AYAs described declining prevalences of depression over time.^26^, ^37^, ^45^, ^55^, ^66^ Ander and colleagues reported a reduction in depression prevalence 10 years after diagnosis.^55^ The other studies recorded lower prevalences at 1,^45^ 6,^37^ and 18 months^66^ from diagnosis and 24 months from surgery.^26^ Contrastingly, Geue and colleagues found no differences in depressive symptomatology among AYA‐CS from baseline (time since diagnosis: within 4 years) to 1 year after.^60^

### 3.7 Predictors

The main reported risk factors associated with a higher prevalence of psychological distress among AYA‐CS were being female^22^, ^25^, ^58^, ^80^, ^84^ having comorbidities and pain,^25^, ^36^, ^50^, ^83^ being unmarried^22^, ^25^, ^33^, ^80^ and being out of school or work.^28^, ^37^, ^38^, ^83^ The most frequently reported risk factor for anxiety was being female.^53^, ^54^, ^55^, ^59^, ^63^, ^70^ Similarly, the risk of depression was higher in females^54^, ^57^, ^70^ and unmarried AYA‐CS.^68^, ^71^

## 4 DISCUSSION

Our systematic review summarized the literature on the worldwide prevalence and risk of psychological distress, anxiety, and depression in AYA‐CS, including longitudinal changes and risk factors based on 68 studies from 16 countries. We estimated that approximately 1 in 3 AYA‐CS were affected by psychological distress or anxiety and 1 in 4 experience depression, with a higher risk in comparison with cancer‐free peers and older cancer survivors. Depressive symptoms seem to decrease over time; however, the findings for psychological distress and anxiety are inconclusive.

### 4.1 Prevalence of psychological distress, anxiety, and depression

We estimated that 32%, 29%, and 24% of AYA‐CS experience psychological distress, anxiety, and depression, respectively. Prior reviews without meta‐analysis have reported a prevalence range between 8% and 41.6% across these psychological outcomes among childhood and AYA cancer survivors with heterogeneous definitions.^10^, ^12^ These prevalences are higher compared to the general population of 15‐39‐year‐olds: About 4% had depression and 5% anxiety disorders in 2019.^91^

We identified possible global variations in the prevalence of psychological distress and anxiety with twofold to threefold higher estimates in Asia and slightly higher in Oceania than in Europe and North America. No studies from Africa and South America were found. Studies conducted in Asia and Oceania used primarily the DT scale (cutoff of 4 or 5) to assess psychological distress and this method reported the highest prevalence rates compared to other assessment tools. No such pattern was seen for anxiety. DT is a fast, one‐item self‐rated distress screener but it might not assess the severity of the distress accurately.^92^ The prevalence of distress for Asia and Oceania might therefore not be comparable to other regions, which were using less sensitive screeners.

Further, cultural variations in the manifestation of mental disorders could impact their prevalence rates, something conventional screening tools might not fully capture.^93^ For instance, within several Asian cultures, the studied mental health issues may predominantly manifest as physical discomfort or somatic conditions rather than the emotional distress more commonly associated with Western cultures.^94^, ^95^ There is uncertainty associated with the accuracy of commonly used screening tools, like HADS for measuring anxiety and DT for psychological distress, in effectively capturing these diverse manifestations among AYAs, even though DT seems to be a sensitive instrument for identifying distress in the studied Asian populations considering the high prevalence estimated for this region.

The treatment status of the participants could also explain the higher rates, since Asian and Oceanian studies recruited majorly on‐treatment cancer survivors for both psychological distress and anxiety. However, for studies reporting on anxiety, no differences were found based on treatment status. Cancer treatment can cause physical, social, and financial challenges for AYAs. Physical (e.g., hair loss and weight fluctuations) and biological changes (e.g., fertility issues) may reduce self‐esteem and affect relationships.^96^, ^97^ Treatment schedules may interfere with education and career goals, leading to financial distress and hindering the establishment of stable functional roles.^97^ These might contribute to generally higher distress during treatment, but specific anxiety and depression symptoms may likely persist beyond treatment timeframes. It remains unclear whether there are actual differences between the geographical regions or to what extent these are influenced by the instruments used, cultural and individual aspects or treatment status of participants.

### 4.2 Risk of developing psychological distress, anxiety, and depression

Most studies found a higher risk of psychological distress, anxiety, and depression when comparing AYA‐CS with older CS^39^, ^42^, ^70^, ^86^ and cancer‐free peers.^22^, ^33^, ^39^, ^43^, ^67^, ^70^, ^88^ Similarly, a prior meta‐analysis of three studies found a 16% higher risk of anxiety and a 36% higher risk of mood disorders among AYAs compared to cancer‐free peers, based on clinical diagnoses.^9^ These differences can be attributed to the complex psychosocial unmet needs of AYA‐CS during a crucial time of life for resource building (e.g., being financially stable, creating intimate relationships, autonomy, and self‐identity). However, the prevalence of unmet needs and other challenges may vary across settings and within AYA populations (e.g., based on age, time since diagnosis, cancer type, and stage), which might explain the negative findings in a few studies. Furthermore, based on a prior review, a substantial proportion of AYAs demonstrate resilience or exhibit post‐traumatic growth, which could account for the absence of discernible differences in psychological distress when compared with their cancer‐free counterparts.^98^

Compared to their siblings, AYAs showed at least a borderline higher risk of anxiety and an increased risk of depression.^44^, ^53^ In contrast, prior studies have shown that siblings also report high levels of distress likely due to shared experiences and unmet needs during cancer treatment or relapse.^99^, ^100^ Future research should take these findings into account when selecting a suitable comparison group to assess mental health outcomes among AYAs.

### 4.3 Psychological outcomes trajectories

Three out of seven studies showed decreased psychological distress in AYAs 12 months after diagnosis and treatment completion,^45^, ^87^, ^89^ while others reported small or no differences.^28^, ^37^, ^52^, ^60^ No changes in anxiety levels were seen in most studies^37^, ^45^, ^60^ while a decrease in depressive symptomatology was reported.^26^, ^37^, ^45^, ^55^, ^66^ These studies scored relatively low in the NOS, with low participant numbers, resulting in limited generalizability to the larger population of AYA‐CS. A previous review found that among ovarian cancer patients, a shorter time since diagnosis was associated with higher distress,^101^ similar to our meta‐analysis findings based on treatment status. However, some AYA‐CS subgroups may experience chronic distress due to difficulties in building resilience, as cancer may be their first exposure to significant life challenges.^98^ Evidence indicates that distress at diagnosis can predict persistent distress,^102^ while higher family and physical functioning can predict lower anxiety and depression levels.^103^ Another systematic review identified anxiety and not depression as a long‐term problem among CS.^104^ Furthermore, the frequently reported fear of cancer recurrence among AYA‐CS^105^ might cause enduring anxiety beyond the initial diagnosis period.

### 4.4 Findings on predictors

Being female was associated with higher levels of psychological distress, anxiety, and depression compared to men across study settings and screening instruments,^22^, ^25^, ^53^, ^54^, ^55^, ^57^, ^58^, ^59^, ^63^, ^70^, ^80^, ^84^ similar to findings from the general population.^106^ These findings should be further explored within age groups of female AYA‐CS, considering that AYA‐CS experience more marital stress and divorce rates compared to controls,^107^ consequences that might disproportionally affect women, who often take more responsibilities in childcare. We found that unmarried AYA‐CS had higher levels of distress and depression compared to their married counterparts.^22^, ^25^, ^33^, ^68^, ^71^, ^80^ Relationships can provide a supportive role (e.g., emotional and financial) during cancer treatment and early survivorship.^107^ Younger AYA‐CS may receive support from peers, which could explain why being out of school and work was also linked to higher distress.^28^, ^37^, ^38^, ^83^

### 4.5 Strengths and limitations

This is the first comprehensive review and meta‐analysis on AYA‐onset cancer survivors estimating the prevalence of psychological distress, anxiety, and depression, including studies using validated screeners and diagnostic interviews.

However, our work has limitations. First, the high heterogeneity between studies concerning outcome assessment and instrument cutoffs, time since diagnosis and treatment status might limit the pooled estimates' generalizability. We addressed this by conducting stratified analyses, where we saw that higher prevalences of psychological distress were reported among AYAs on treatment and where the DT was used, higher anxiety among Asian AYAs and no differences related to depression prevalence. The pooled estimates should be interpreted considering the different subgroups within the AYA population. Other factors such as cultural or clinical might explain the remaining heterogeneity which could not be explored in this work and need to be addressed by future studies. Second, the infrequently used instruments were grouped as “other,” limiting interpretation. Additionally, we were unable to retrieve six studies that could have potentially contributed to a higher statistical power in our meta‐analysis. However, it is unclear whether these studies would have met our inclusion or quality criteria. Given that these unretrieved studies constitute only 2% of all the studies we screened in full‐text, likely their absence did not impact our results. Another limitation is the potential publication bias. This might have led to an overestimation of the anxiety prevalence. For depression, after exclusion of the unsatisfactory quality studies, no publication bias was detected, which might reflect any methodological limitations of studies rather than actual publication bias. Lastly, we included data on AYA‐CS from stratified analyses; however, the search strategy was not built to capture all studies, which may have included a subgroup of CS within the AYA age range.

### 4.6 Future directions

Oncology services should incorporate mental health screening starting at cancer diagnosis, in order to detect psychological distress early and prevent possible future complications, while a continued psychological care should be a fundamental part of the countries' cancer survivorship models. Larger prospective studies using validated instruments and if possible clinical interviews for a subset of their participants should be conducted, to fully assess the risk and trajectories of psychological distress, anxiety, and depression in AYA‐CS. Studies should investigate the validity of commonly used instruments for diagnosing anxiety and depression among AYAs across settings. Population‐based or cancer registry‐based prevalence studies should be conducted in African and South American countries so evidence‐based care can be provided for AYA‐CS in these populations. Similarly, potential geographical variations should be further explored to examine any possible differences in unmet needs among AYAs and related cultural aspects in the manifestation of mental health disorders. Age‐appropriate randomized control trials should be conducted in AYA‐CS to identify effective interventions for preventing distress, anxiety, and depression or alleviating their symptomatology as well as efforts should be made to strategize the implementation of preventive and treatment interventions in real‐world settings. This implementation should take into consideration the inclusion of different healthcare professionals, including social workers, psychologists, and psychiatrists, which are trained in AYA issues, considering their unique needs. Additionally, it should recognize the significant role of family members and peer groups in managing mental health disorders among AYAs. Risk factors should be further explored, considering possible interactions between them and within AYA subgroups.

## 5 CONCLUSIONS

Based on our comprehensive review and meta‐analyses, we found that a considerable number of AYA‐CS are experiencing psychological distress, anxiety, and depression. Our findings identify AYAs with cancer as a group at risk of mental health disorders, where interventions should be directed considering that psychological conditions such as anxiety and depression are associated with a 27% heightened risk of cancer mortality among cancer survivors.^108^ Mental health screening should be an integral part of cancer care services as well as age‐ and culturally appropriate interventions should be available to AYA‐CS.

## AUTHOR CONTRIBUTIONS

**Vanesa Osmani:** Conceptualization (lead); data curation (equal); formal analysis (lead); investigation (equal); methodology (lead); project administration (lead); software (equal); validation (equal); visualization (lead); writing – original draft (lead); writing – review and editing (equal). **Lucy Hörner:** Investigation (equal); validation (supporting); writing – review and editing (supporting). **Stefanie J. Klug:** Methodology (supporting); resources (lead); supervision (supporting); writing – review and editing (equal). **Luana Fiengo Tanaka:** Conceptualization (supporting); methodology (supporting); project administration (supporting); supervision (lead); validation (equal); writing – original draft (supporting); writing – review and editing (equal).

## FUNDING INFORMATION

This research received no specific grant from any funding agency, commercial or not‐for‐profit sectors.

## CONFLICT OF INTEREST STATEMENT

The authors declare none.

## ETHICS STATEMENT

This work did not involve human or animal subjects; therefore, no ethics approval or informed consent was necessary.

## Supporting information


**Data S1:** Supporting Information

## Data Availability

All the data which were used for this study can be found in the online supplement.
