# Supplementary material for: Prevalence and risk of psychological distress, anxiety and depression in adolescent and young adult (AYA) cancer survivors: A systematic review and meta‐analysis
Source: Cancer Med. 2023 Aug 10;12(17):18354–67. doi: 10.1002/cam4.6435 (PMC10523984; doi:10.1002/cam4.6435)
Supplement: Supplementary file 1 — Data S1: Supporting Information [file CAM4-12-18354-s001.pdf]

SUPPORTING MATERIAL

Prevalence and Risk of Psychological Distress, Anxiety and Depression in Adolescent and Young Adult (AYA) Cancer Survivors: A Systematic Review and Meta-Analysis

V. Osmani, M.Sc., L. Hörner, B.Sc., Prof. Dr. SJ. Klug, Dr. L. Fiengo Tanaka

eFigure 1. Modified Newcastle-Ottawa scale for assessing the quality of longitudinal studies.

eFigure 2. Modified Newcastle-Ottawa scale for assessing the quality of cross-sectional studies.

eFigure 3. Meta-analysis results on prevalence of psychological distress among AYA cancer survivors by geographical region (30 studies; 15213 participants).

eFigure 4. Meta-analysis results on prevalence of psychological distress among AYA cancer survivors by treatment status (30 studies; 15213 participants).

eFigure 5. Meta-analysis results on prevalence of psychological distress among AYA cancer survivors by instrument and cut-off (30 studies; 15213 participants).

eFigure 6. Meta-analysis results on prevalence of anxiety among AYA cancer survivors by geographical region (24 studies; 8751 participants).

eFigure 7. Meta-analysis results on prevalence of anxiety among AYA cancer survivors by treatment status (24 studies; 8751 participants).

eFigure 8. Meta-analysis results on prevalence of anxiety among AYA cancer survivors by assessment instrument and cut-off (24 studies; 8751 participants).

eFigure 9. Meta-analysis results on prevalence of depression among AYA cancer survivors by geographical region (35 studies; 16638 participants).

eFigure 10. Meta-analysis results on prevalence of depression among AYA cancer survivors by treatment status (35 studies; 16638 participants).

eFigure 11. Meta-analysis results on prevalence of depression among AYA cancer survivors by instrument and cut-off (35 studies; 16638 participants).

eFigure 12. Odds ratios (OR) and corresponding 95% confidence intervals (95%CI) investigating psychological distress among AYAs in comparison to older cancer survivors (1 study) and cancer-free peers (3 studies).

eFigure 13. Risk ratios (OR) and corresponding 95% confidence intervals (95%CI) investigating psychological distress among AYAs in comparison to older cancer survivors and cancer-free peers (1 study).

eFigure 14. Odds ratios (OR) and corresponding 95% confidence intervals (95%CI) investigating anxiety disorders among AYAs in comparison to older cancer survivors (4 studies) and cancer-free peers or siblings (2 studies).

eFigure 15. Risk ratios (OR) and corresponding 95% confidence intervals (95%CI) investigating anxiety disorders among AYAs in comparison to cancer-free peers (1 study) and siblings (1 study).

eFigure 16. Odds ratios (OR) and corresponding 95% confidence intervals (95%CI) investigating depression and mood disorders among AYAs in comparison to younger cancer survivors (1 study) older cancer survivors (3 studies) and cancer-free peers or siblings (3 studies).

eFigure 17. Risk ratios (RR) and corresponding 95% confidence intervals (95%CI) investigating depression and mood disorders among AYAs in comparison to cancer-free peers (3 studies) and siblings (2 studies).

eFigure 18. Meta-analysis results on prevalence of psychological distress among AYA cancer survivors (excluding studies with unsatisfactory ratings)

eFigure 19. Meta-analysis results on prevalence of psychological distress among AYA cancer survivors by geographical region (excluding studies with unsatisfactory ratings)

eFigure 20. Meta-analysis results on prevalence of psychological distress among AYA cancer survivors by treatment status (excluding studies with unsatisfactory ratings)

eFigure 21. Meta-analysis results on prevalence of psychological distress among AYA cancer survivors by instrument (excluding studies with unsatisfactory ratings)

eFigure 22. Meta-analysis results on prevalence of anxiety among AYA cancer survivors (excluding studies with unsatisfactory ratings)

eFigure 23. Meta-analysis results on prevalence of anxiety among AYA cancer survivors by geographical region (excluding studies with unsatisfactory ratings)

eFigure 24. Meta-analysis results on prevalence of anxiety among AYA cancer survivors by treatment status (excluding studies with unsatisfactory ratings)

eFigure 25. Meta-analysis results on prevalence of anxiety among AYA cancer survivors by instrument (excluding studies with unsatisfactory ratings)

eFigure 26. Meta-analysis results on prevalence of depression among AYA cancer survivors (excluding studies with unsatisfactory ratings)

eFigure 27. Meta-analysis results on prevalence of depression among AYA cancer survivors by geographical region (excluding studies with unsatisfactory ratings)

eFigure 28. Meta-analysis results on prevalence of depression among AYA cancer survivors by treatment status (excluding studies with unsatisfactory ratings)

eFigure 29. Meta-analysis results on prevalence of depression among AYA cancer survivors by instrument (excluding studies with unsatisfactory ratings)

eTable 1. Eligibility criteria according to the PECOS schema.

eTable 2. Search strategy used to identify eligible studies.

eTable 3. Selected characteristics of the included studies and quality assessment results (n= 68).

eTable 4. Newcastle-Ottawa quality assessment scoring for the 68 included studies.

eTable 5. Main findings on prevalence of psychological distress, anxiety and depression in AYA cancer survivors, comparison with reference groups and predictors

eTable 6. Main findings on risk of developing psychological distress, anxiety and depression in AYA cancer survivors (n=16)

eTable 7. Main findings on trajectories of psychological distress, anxiety and depression among AYA cancer survivors (n=10)

MODIFIED NEWCASTLE - OTTAWA QUALITY ASSESSMENT SCALE
COHORT STUDIES

Selection (Maximum 5 stars)

1) Representativeness of the exposed cohort
   a) truly representative of the average in the target population ❋ (all subjects or random sampling)
   b) somewhat representative of the average in the target population ❋ (non-random sampling)
   c) selected group of users/single institutions
   d) no description of the derivation of the cohort

2) Selection of the non-exposed cohort
   a) drawn from the same community as the exposed cohort ❋
   b) drawn from a different source
   c) no description of the derivation of the non-exposed cohort

3) Ascertainment of exposure (cancer diagnosis)
   a) cancer registry/clinical registry/hospital records ❋❋
   b) self-report ❋
   d) no description

4) Demonstration that outcome of interest was not present at start of study
   a) yes ❋
   b) no

Comparability (Maximum 2 stars)

1) Comparability of cohorts on the basis of the design or analysis
   a) study controls for sex ❋
   b) study controls for any additional factor ❋ (e.g. treatment, time since diagnosis, current age, prior mental health disorders)

Outcome (Maximum 3 stars)

1) Assessment of outcome
   a) clinical diagnosis based on DSM criteria ❋❋
   b) record linkage ❋❋
   c) validated screeners ❋
   d) no description

2) Adequacy of follow up of cohorts
   a) complete and long enough follow up - all subjects accounted for ❋
   b) subjects lost to follow up unlikely to introduce bias - small number lost - > 20% follow up, or description provided of those lost) ❋
   c) follow up not long enough for outcomes to occur and rate <20% and no description of those lost
   d) no statement

eFigure 1. Modified Newcastle-Ottawa scale for assessing the quality of longitudinal studies

Note: A study was considered representative if participants were recruited through random sampling strategies, cancer registries and databases or from multiple clinics or hospitals. Regarding exposure assessment, studies were assigned two stars if the participants were identified through cancer registries, medical records and databases and one star if they self-reported their diagnosis. Studies were assigned one star if they used validated tools (e.g. screeners) to assess psychological distress, anxiety and depression and two stars if the AYAs were clinically diagnosed with any of the outcomes of interest based on the DSM criteria for mental disorders. If no non-exposed cohort was selected, it was identified as no comparability between cohorts in terms of the variables involved and the study got no stars.

MODIFIED NEWCASTLE - OTTAWA QUALITY ASSESSMENT SCALE ADAPTED FOR CROSS-SECTIONAL STUDIES

Selection (Maximum 5 stars):

1) Representativeness of the sample:

a) truly representative of the average in the target population. ❋ (all subjects or random sampling)
b) somewhat representative of the average in the target population. ❋ (non-random sampling)
c) selected group of users
d) no description of the sampling strategy

2) Sample size:

a) justified and satisfactory ❋
b) not justified

3) Non-respondents:

a) comparability between respondents and non-respondents characteristics is established, and the response rate is satisfactory ❋
b) the response rate is unsatisfactory, or the comparability between respondents and non-respondents is unsatisfactory
c) no description of the response rate or the characteristics of the responders and the non-responders

4) Ascertainment of the exposure (cancer diagnosis):

a) cancer registry/clinical registry/hospital records ❋❋
b) self-report ❋
c) no description of the measurement tool

Comparability (Maximum 2 stars):

1) The subjects in different outcome groups are comparable, based on the study design or analysis. Confounding factors are controlled.

a) the study controls for the most important factor (sex) ❋
b) the study control for any additional factor (e.g. treatment, time since diagnosis, current age, prior mental health disorders)❋

Outcome (Maximum 3 stars):

1) Assessment of outcome:

a) clinical diagnosis based on DSM criteria ❋❋
b) record linkage ❋❋
c) validated screeners ❋
d) self-report or no description

2) Statistical test:

a) the statistical test used to analyze the data is clearly described and appropriate, and the measurement of the association is presented, including confidence intervals and the probability level (p value). ❋
b) the statistical test is not appropriate, not described or incomplete.

eFigure 2. Modified Newcastle-Ottawa scale for assessing the quality of cross-sectional studies.

Note: A study was considered representative if participants were recruited through random sampling strategies, cancer registries and databases or from multiple clinics or hospitals. Regarding exposure assessment, studies were assigned two stars if the participants were identified through cancer registries, medical records and databases and one star if they self-reported their diagnosis. A response rate of 70% or more was considered as satisfactory. A study was assigned a star if additionally, reported data regarding differences between respondents and non-respondents. Studies were assigned one star if they used validated tools (e.g. screeners) to assess psychological distress, anxiety and depression and two stars if the AYAs were clinically diagnosed with any of the outcomes of interest based on the DSM criteria for mental disorders.

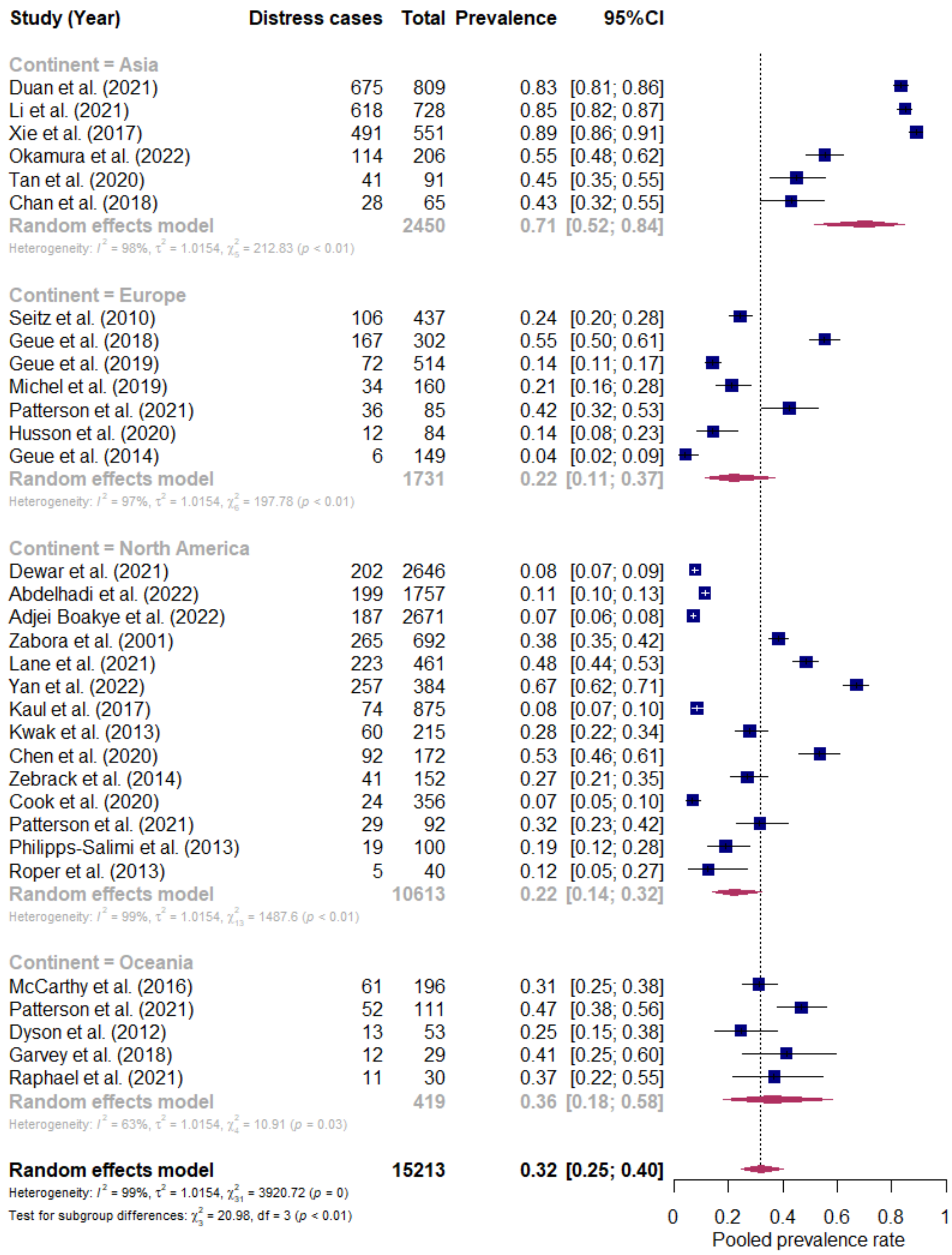

eFigure 3. Meta-analysis results on prevalence of psychological distress among AYA cancer survivors by geographical region (30 studies; 15213 participants).

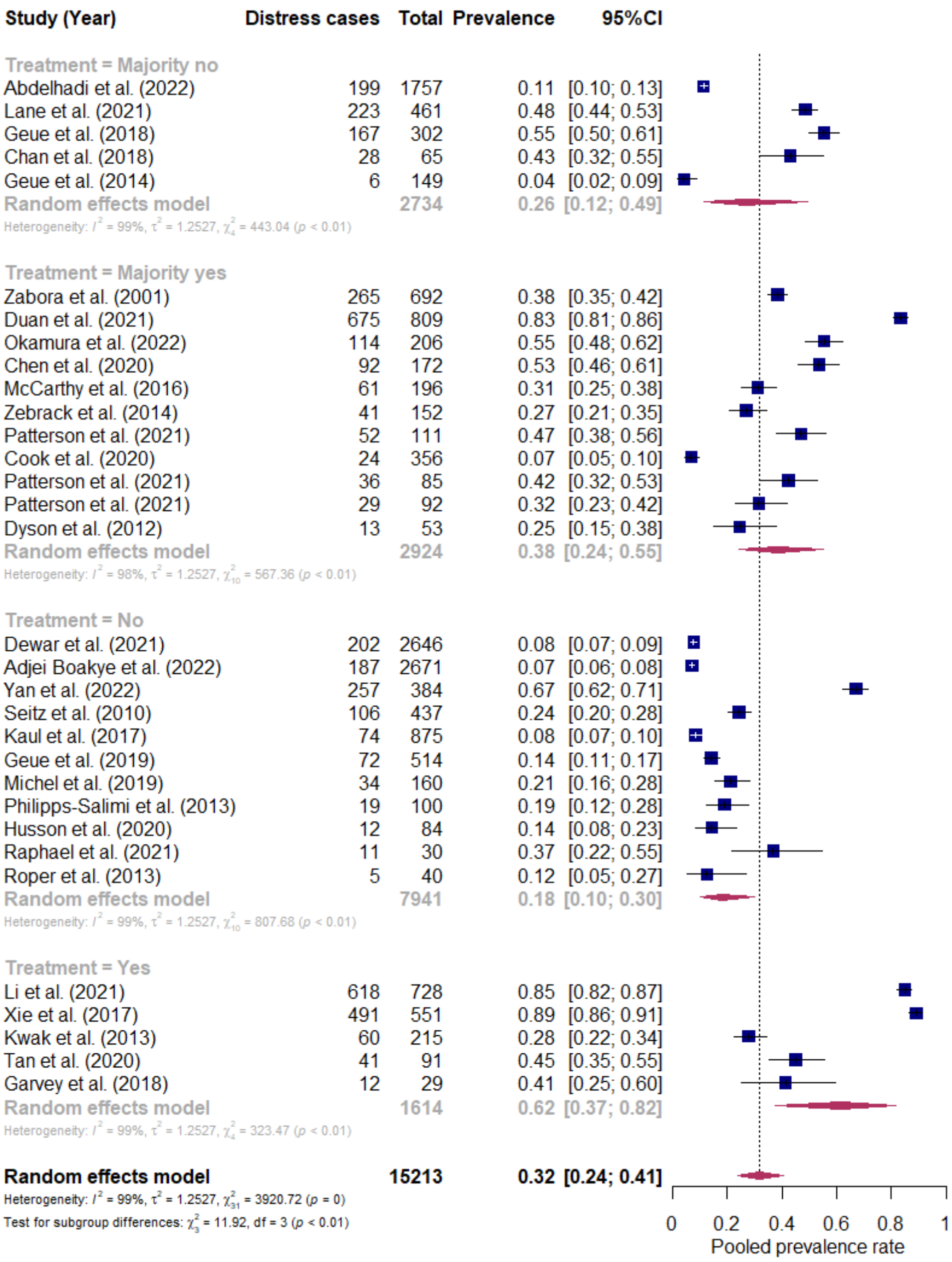

eFigure 4. Meta-analysis results on prevalence of psychological distress among AYA cancer survivors by treatment status (30 studies; 15213 participants).

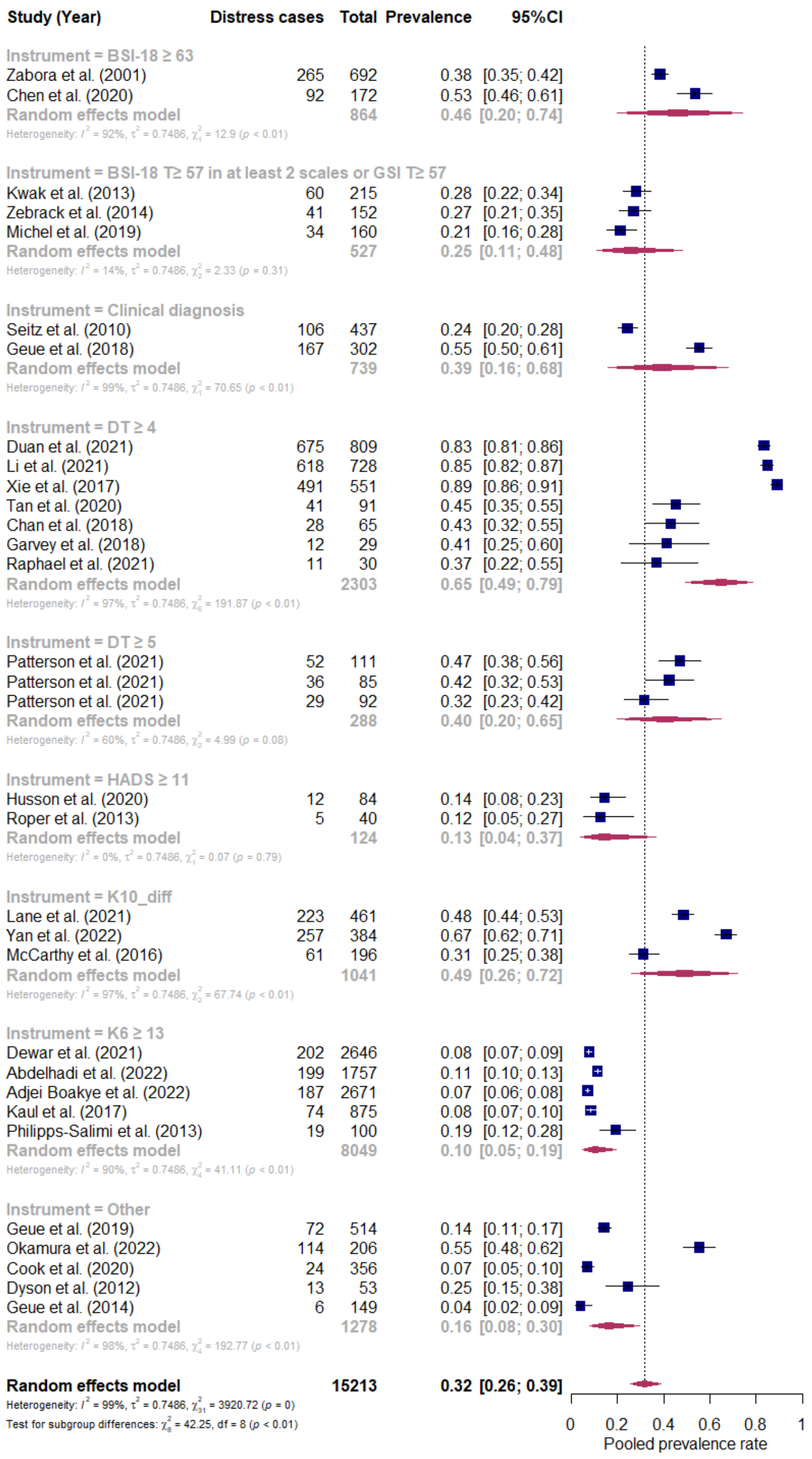

eFigure 5. Meta-analysis results on prevalence of psychological distress among AYA cancer survivors by instrument and cut-off (30 studies; 15213 participants).

[K10_diff includes studies using the K10 instrument with different cut-offs; in the “Other” group, studies using infrequently used instruments were grouped together]

BSI-18, Brief Symptom Inventory – 18; DT, Distress Thermometer; HADS, The Hospital Anxiety and Depression Scale; K10, The Kessler Psychological Distress Scale – 10; K6, The Kessler Psychological Distress Scale – 6.

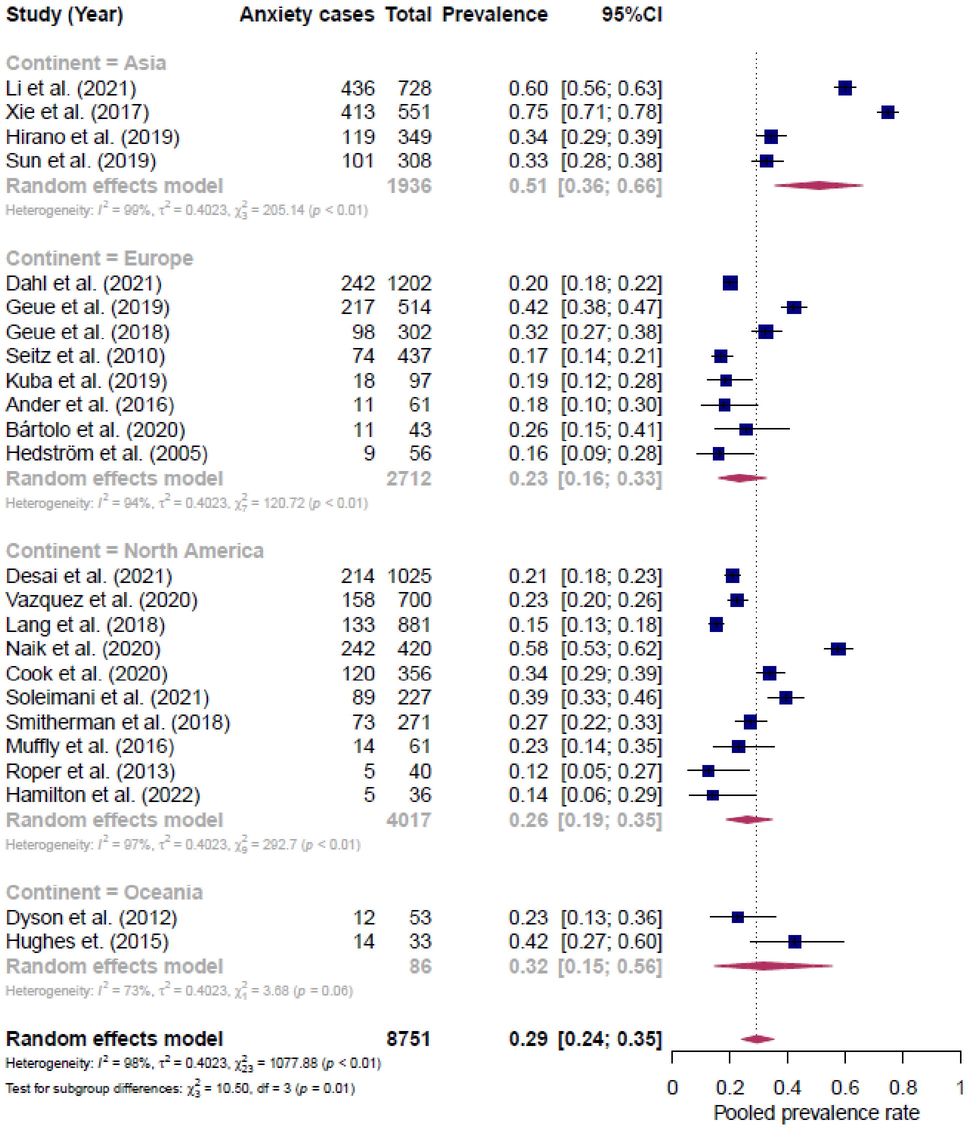

eFigure 6. Meta-analysis results on prevalence of anxiety among AYA cancer survivors by geographical region (24 studies; 8751 participants).

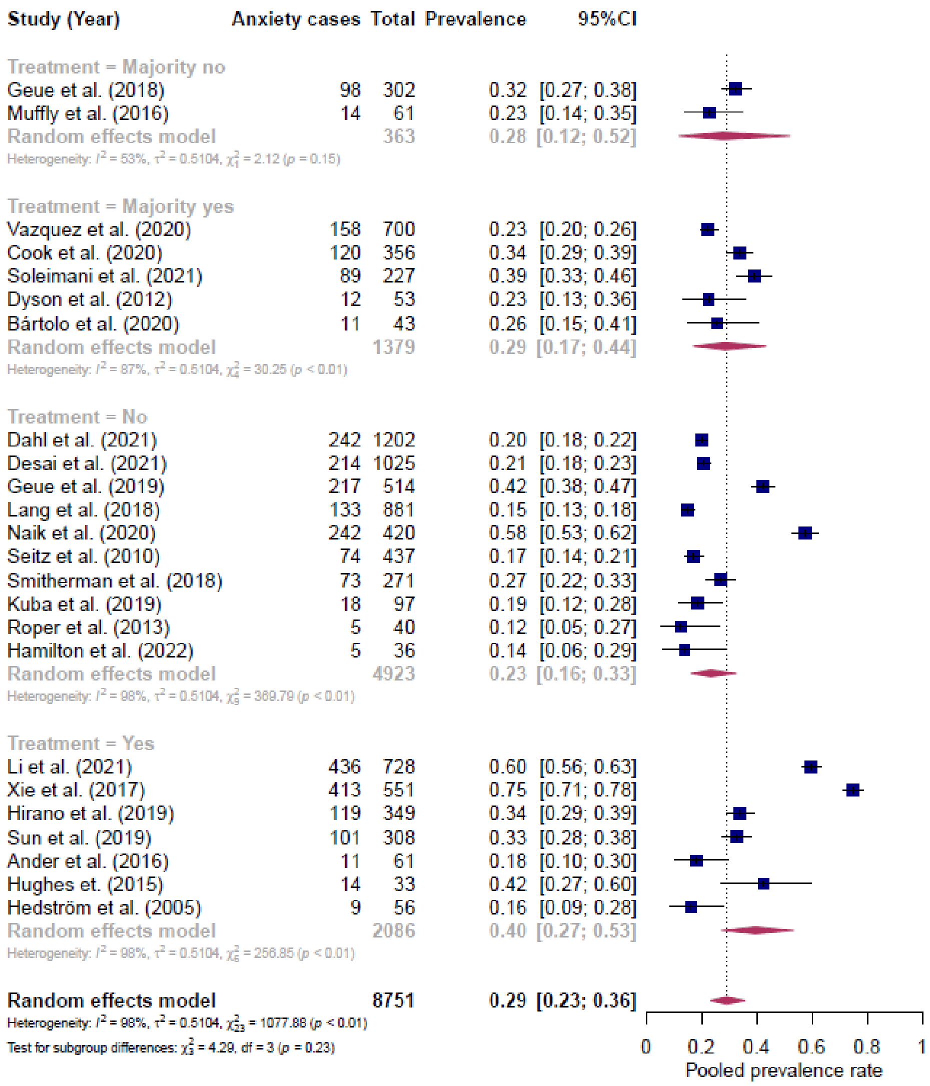

eFigure 7. Meta-analysis results on prevalence of anxiety among AYA cancer survivors by treatment status (24 studies; 8751 participants).

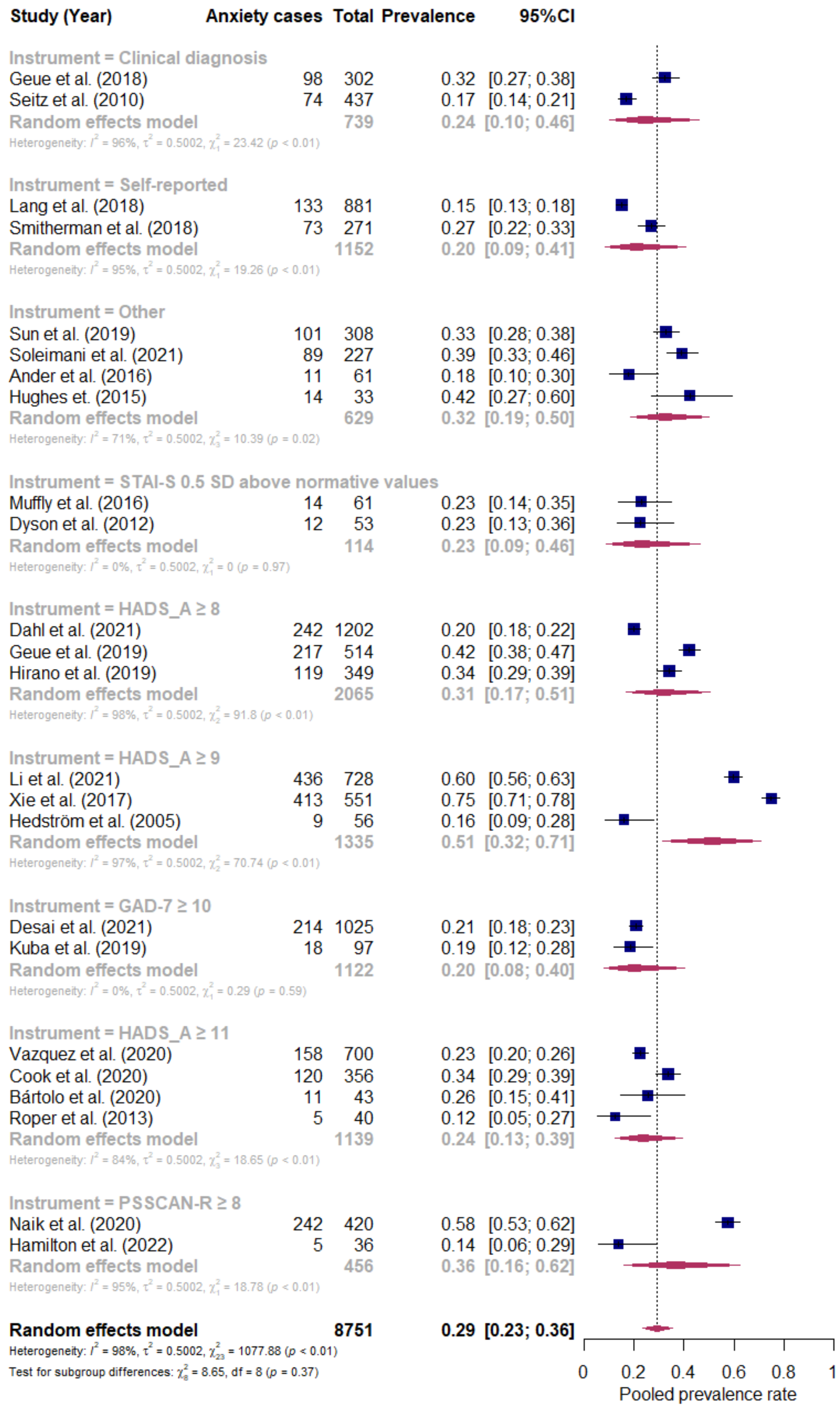

eFigure 8. Meta-analysis results on prevalence of anxiety among AYA cancer survivors by assessment instrument and cut-off (24 studies; 8751 participants).

[Self-reported includes studies where participants self-reported a professionally diagnosed anxiety disorder; in the “Other” group, studies using infrequently used instruments were grouped together]

SD, standard deviation; STAI, State-Trait Anxiety Inventory; HADS, The Hospital Anxiety and Depression Scale; GAD-7, Generalized Anxiety Disorder Scale - 7; PSSCAN-R, The Psychosocial Screen for Cancer.

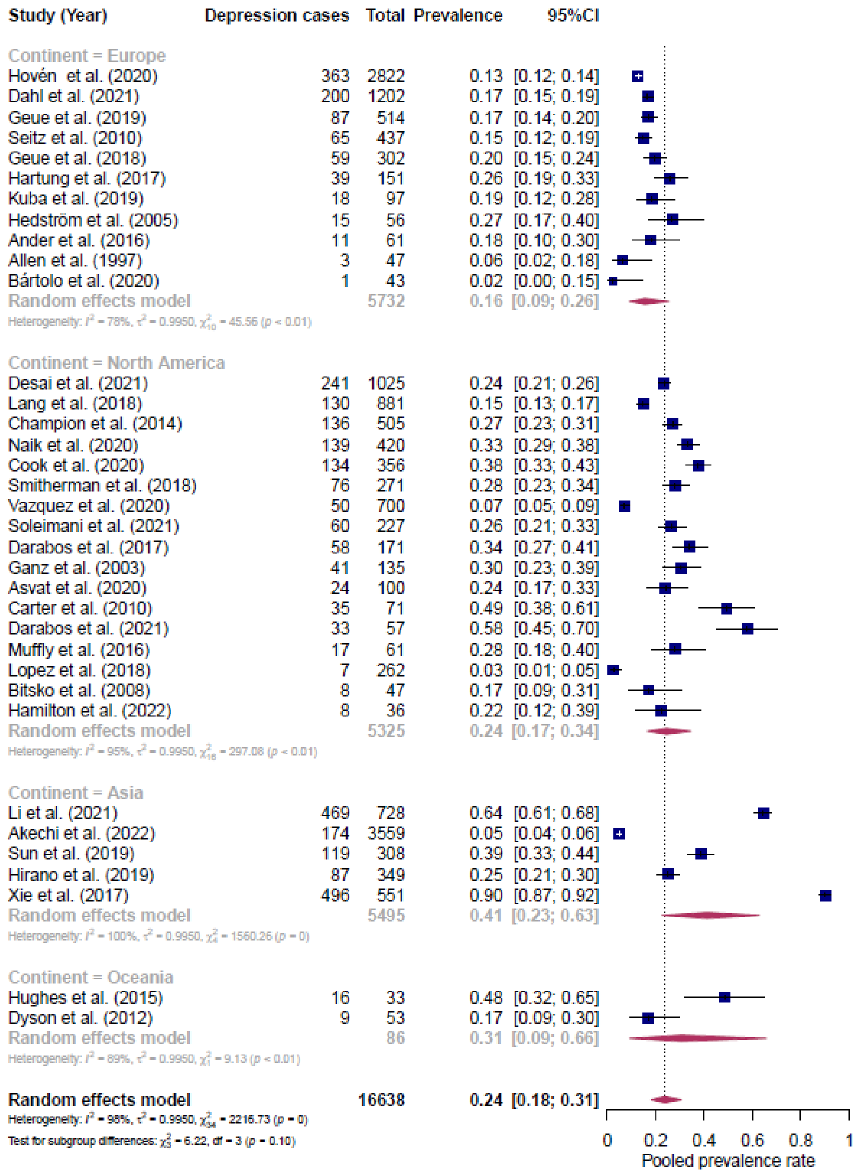

eFigure 9. Meta-analysis results on prevalence of depression among AYA cancer survivors by geographical region (35 studies; 16638 participants).

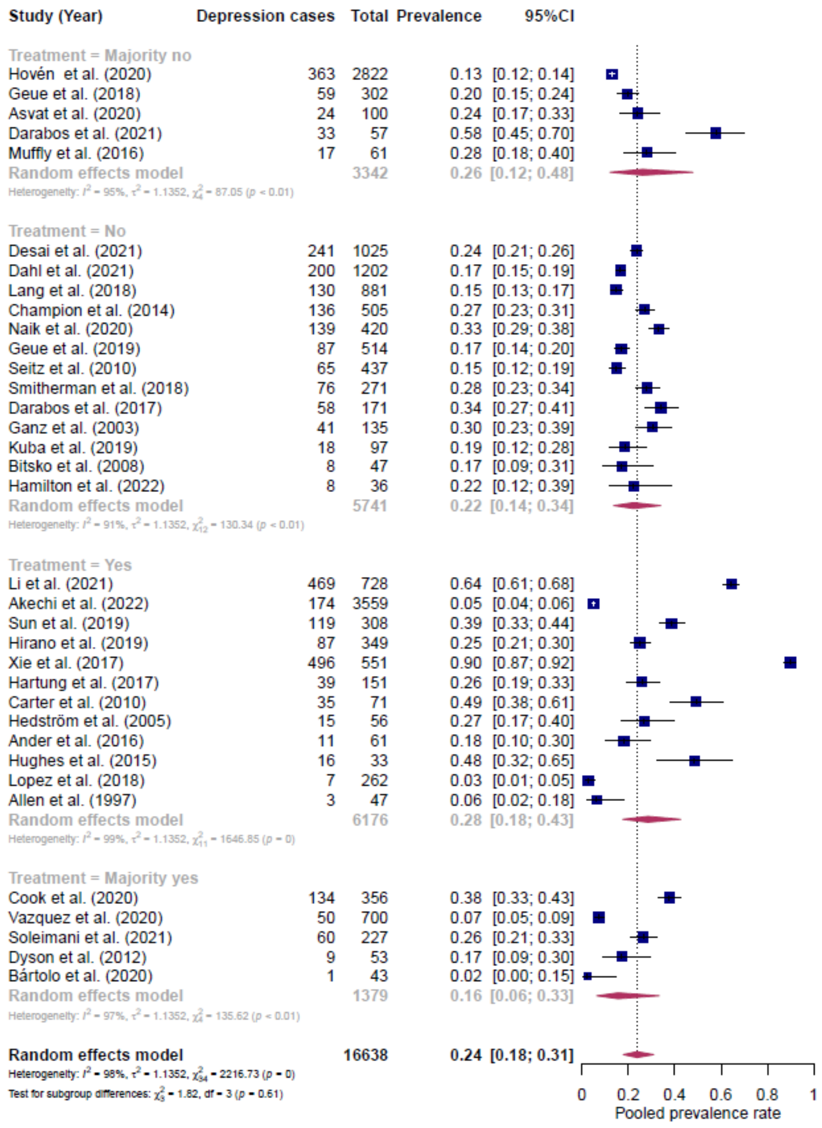

eFigure 10. Meta-analysis results on prevalence of depression among AYA cancer survivors by treatment status (35 studies; 16638 participants).

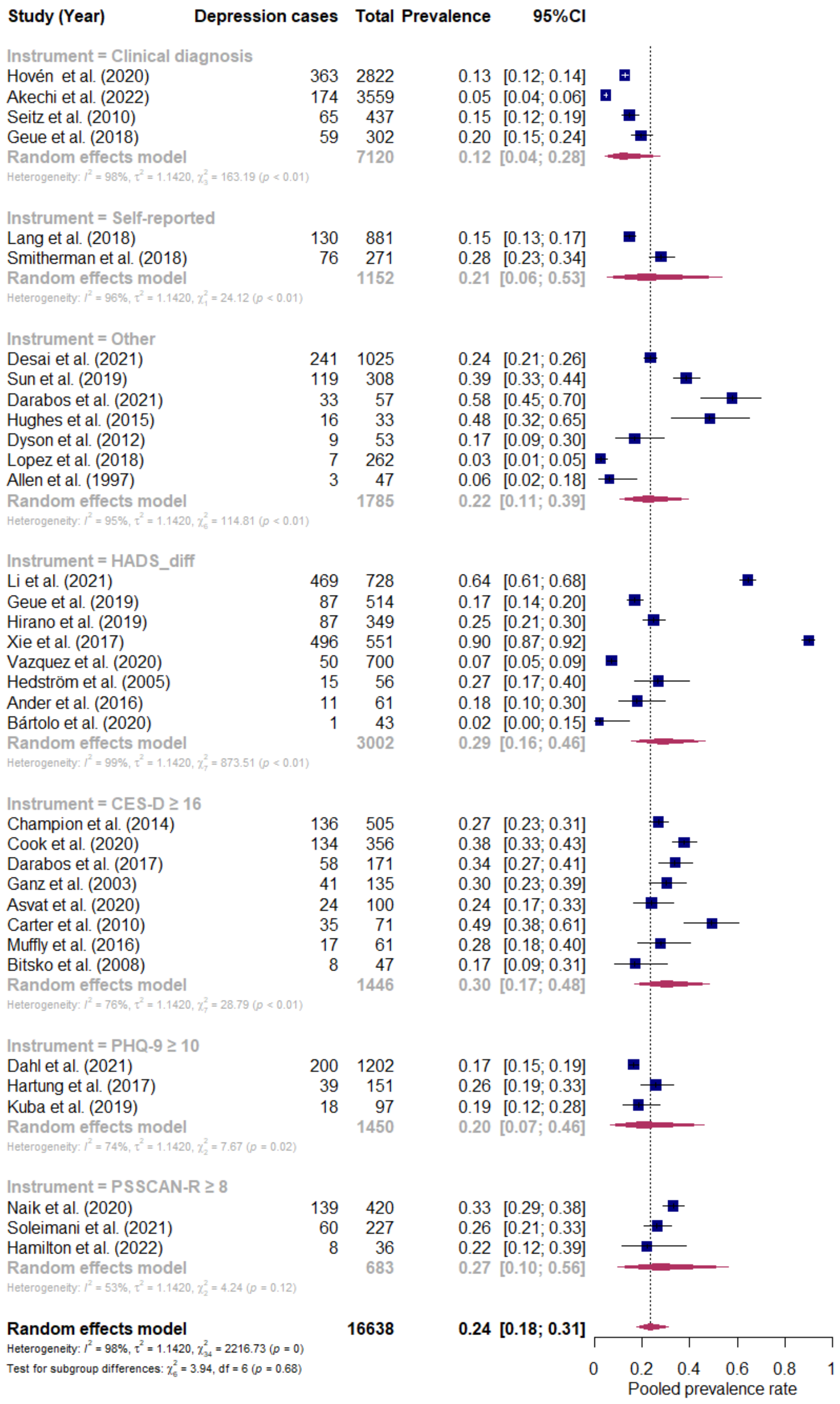

eFigure 11. Meta-analysis results on prevalence of depression among AYA cancer survivors by instrument and cut-off (35 studies; 16638 participants).

[Self-reported includes studies where participants self-reported a professionally diagnosed depressive/mood disorder; in the "Other" group, studies using infrequently used instruments were grouped together; HADS_diff includes studies using the HADS instrument with different cut-offs]

HADS, The Hospital Anxiety and Depression Scale; CES-D, Center for Epidemiologic Studies Depression Scale; PHQ-9, Patient Health Questionnaire-9; PSSCAN-R, The Psychosocial Screen for Cancer.

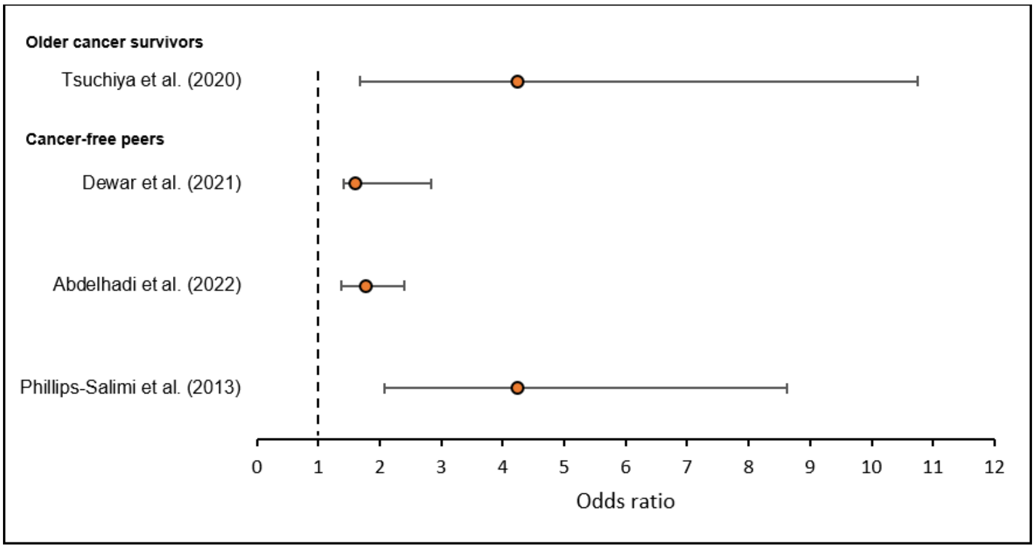

eFigure 12. Odds ratios (OR) and corresponding 95% confidence intervals (95%CI) investigating psychological distress among AYAs in comparison to older cancer survivors (1 study) and cancer-free peers (3 studies).

[Older cancer survivors were those diagnosed with cancer after 39 years of age]

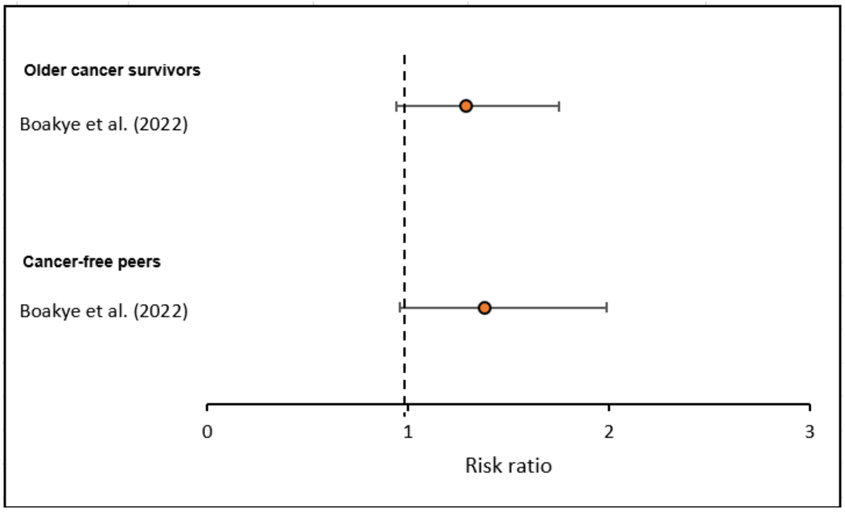

eFigure 13. Risk ratios (OR) and corresponding 95% confidence intervals (95%CI) investigating psychological distress among AYAs in comparison to older cancer survivors and cancer-free peers (1 study).

[The same study compared AYA with older cancer survivors and cancer-free peers; Older cancer survivors were those diagnosed with cancer after 39 years of age]

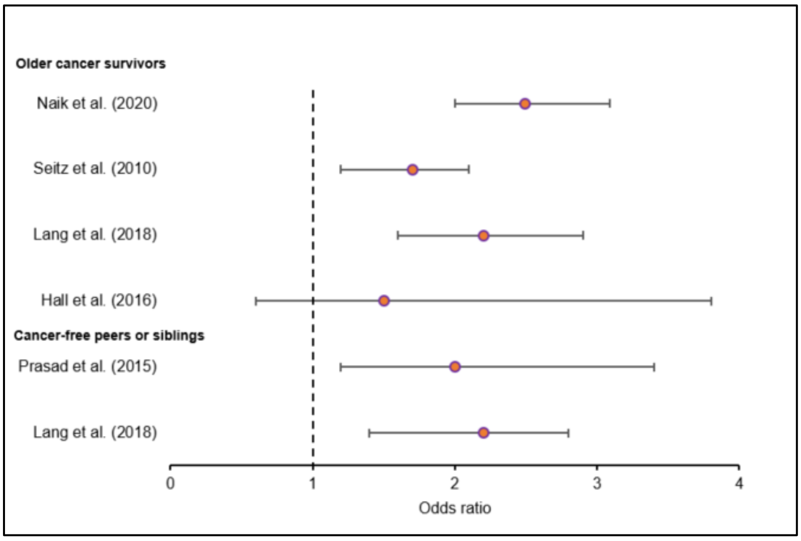

eFigure 14. Odds ratios (OR) and corresponding 95% confidence intervals (95%CI) investigating anxiety disorders among AYAs in comparison to older cancer survivors (4 studies) and cancer-free peers or siblings (2 studies).

[Older cancer survivors were those diagnosed with cancer after 39 years of age]

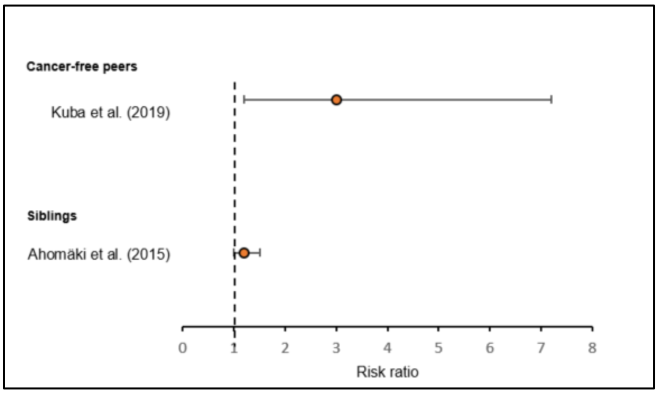

eFigure 15. Risk ratios (OR) and corresponding 95% confidence intervals (95%CI) investigating anxiety disorders among AYAs in comparison to cancer-free peers (1 study) and siblings (1 study).

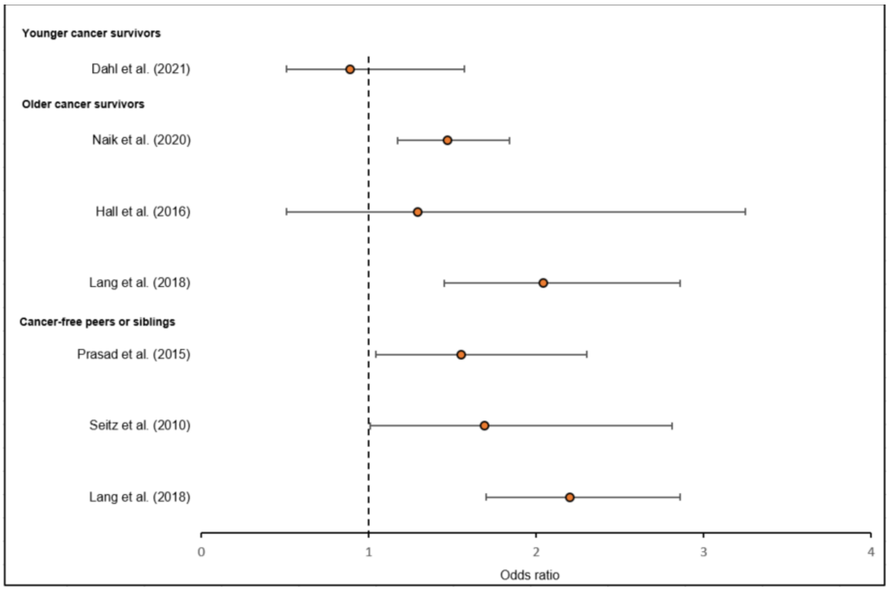

eFigure 16. Odds ratios (OR) and corresponding 95% confidence intervals (95%CI) investigating depression and mood disorders among AYAs in comparison to younger cancer survivors (1 study) older cancer survivors (3 studies) and cancer-free peers or siblings (3 studies).

[Older cancer survivors were those diagnosed with cancer after 39 years of age; younger cancer survivors were those diagnosed with cancer during childhood]

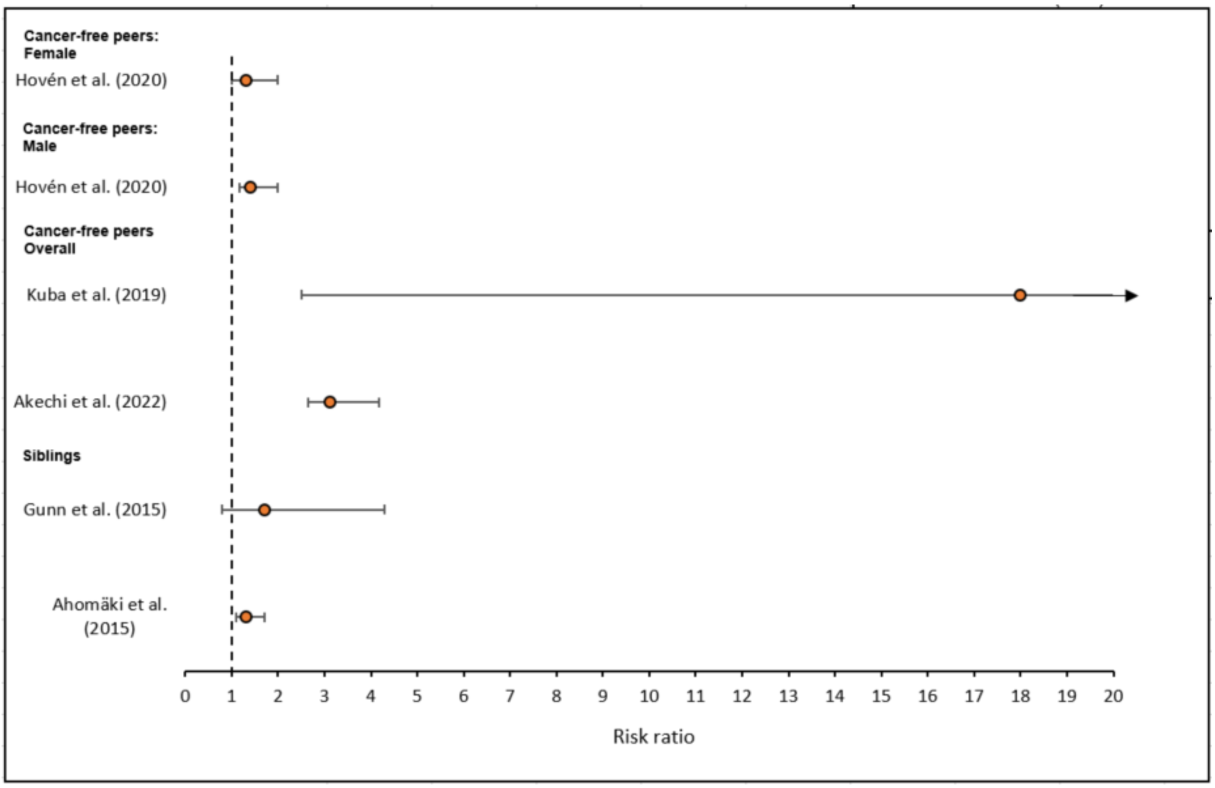

eFigure 17. Risk ratios (RR) and corresponding 95% confidence intervals (95%CI) investigating depression and mood disorders among AYAs in comparison to cancer-free peers (3 studies) and siblings (2 studies).

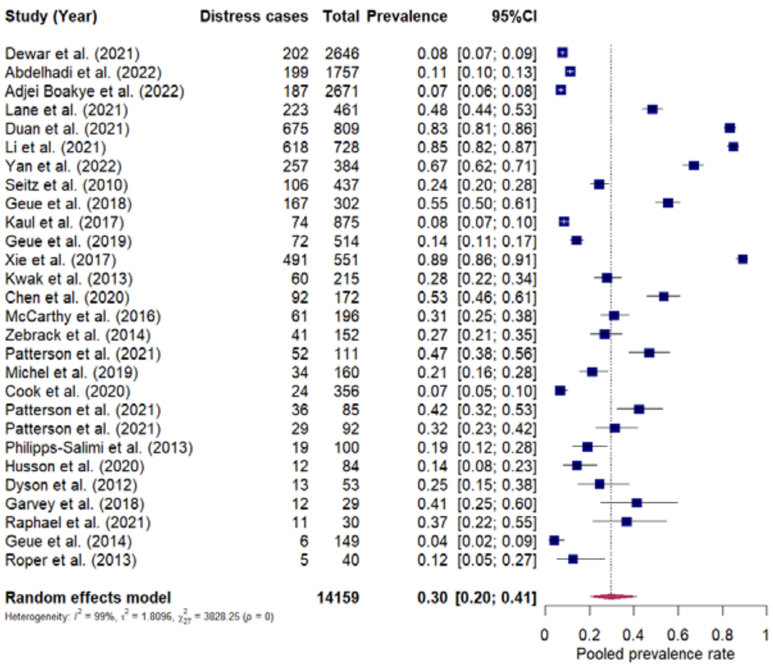

eFigure 18. Meta-analysis results on prevalence of psychological distress among AYA cancer survivors (excluding studies with unsatisfactory ratings)

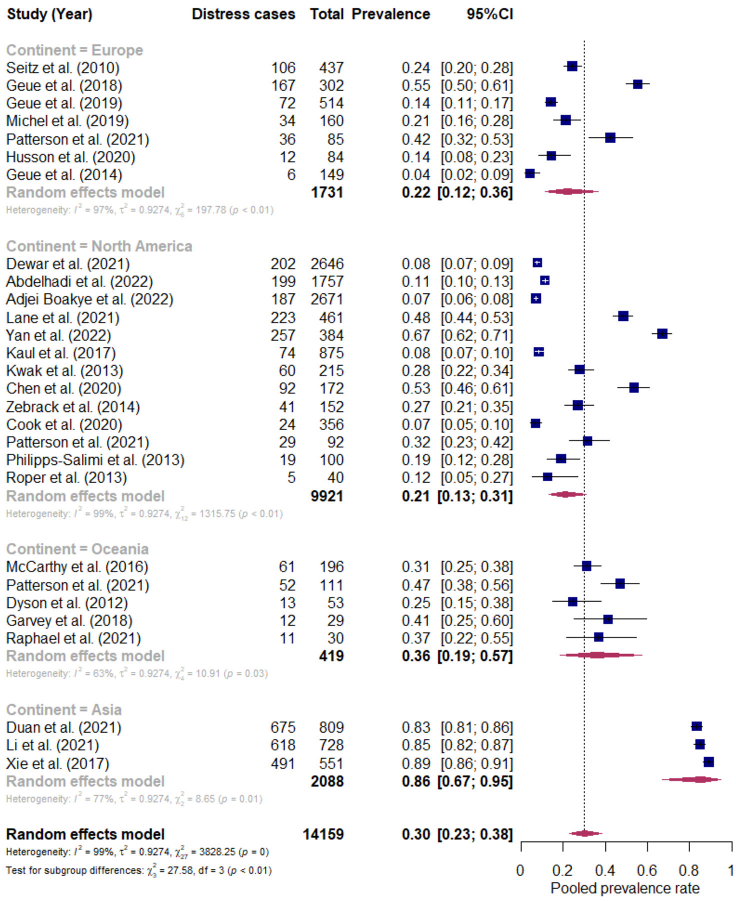

eFigure 19. Meta-analysis results on prevalence of psychological distress among AYA cancer survivors by geographical region (excluding studies with unsatisfactory ratings)

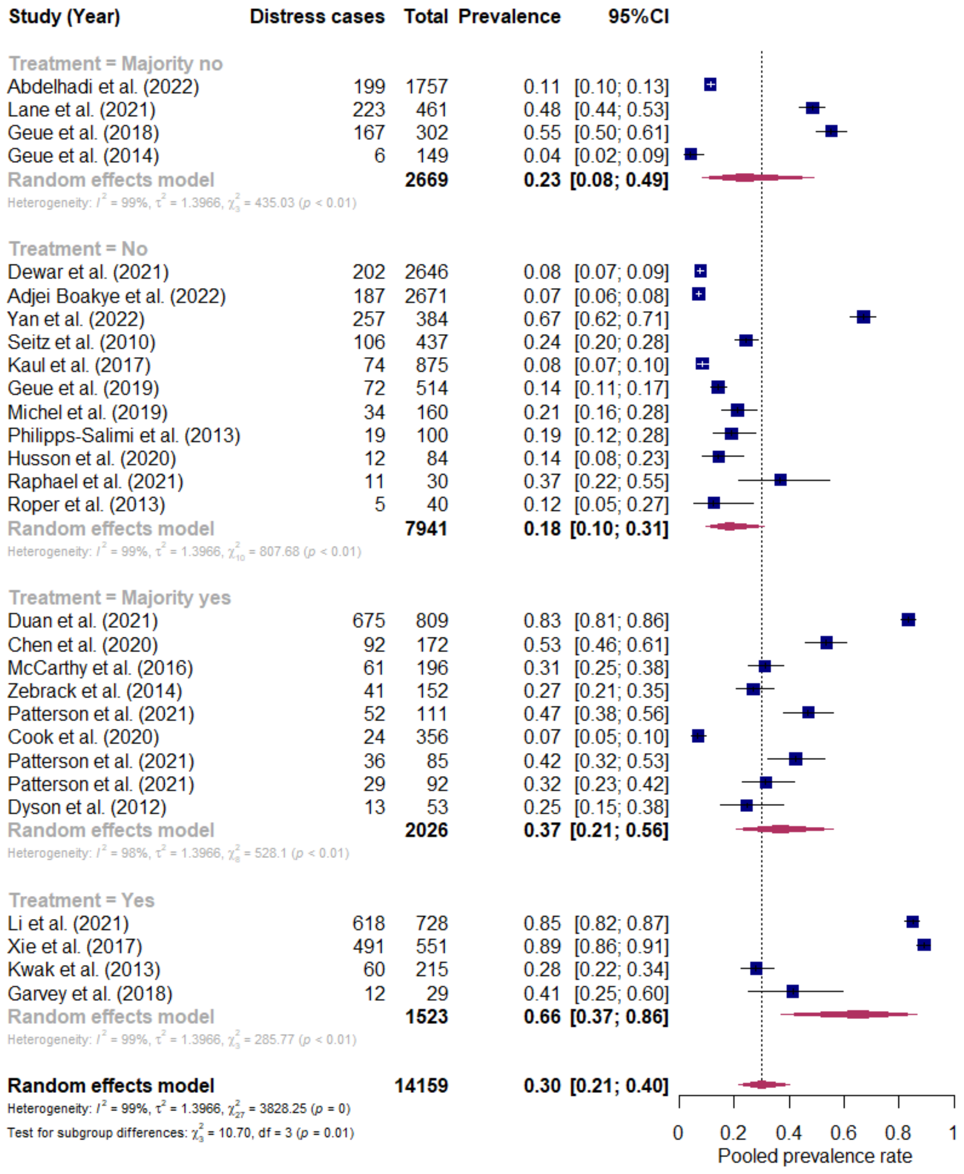

eFigure 20. Meta-analysis results on prevalence of psychological distress among AYA cancer survivors by treatment status (excluding studies with unsatisfactory ratings)

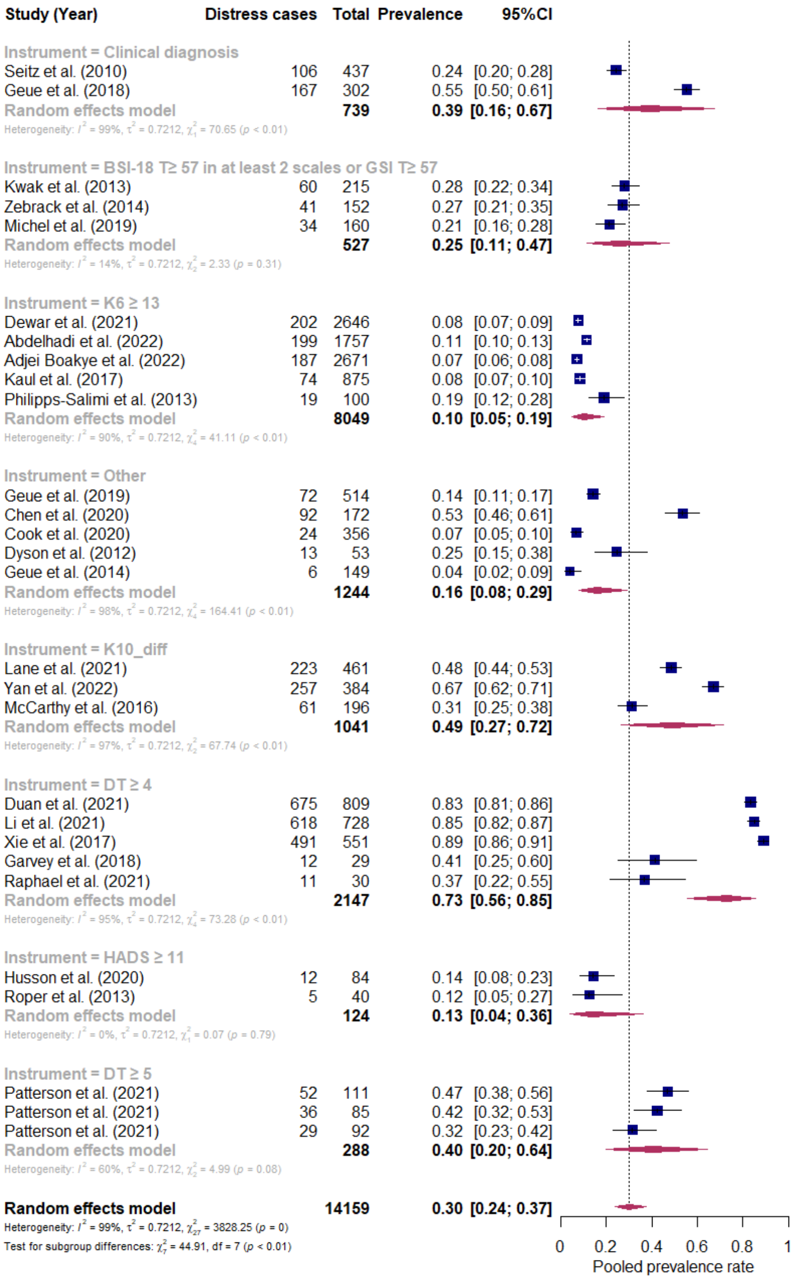

eFigure 21. Meta-analysis results on prevalence of psychological distress among AYA cancer survivors by instrument (excluding studies with unsatisfactory ratings)

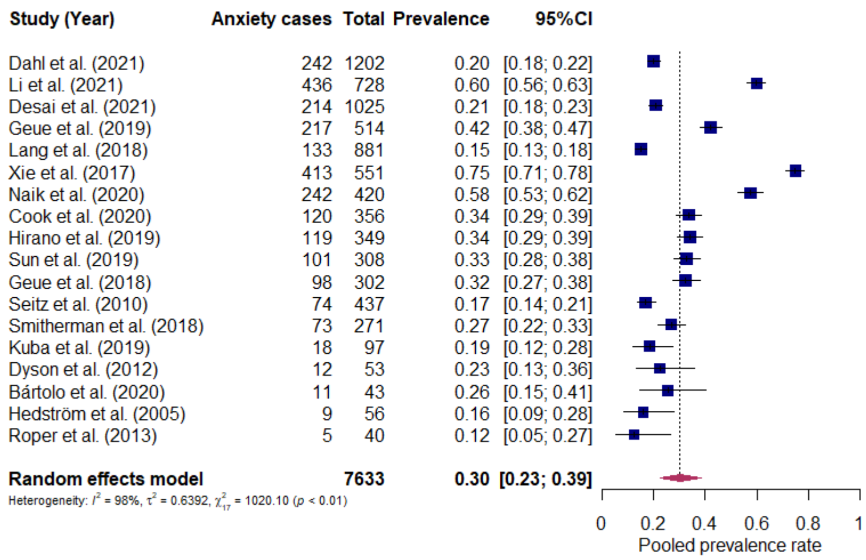

eFigure 22. Meta-analysis results on prevalence of anxiety among AYA cancer survivors (excluding studies with unsatisfactory ratings)

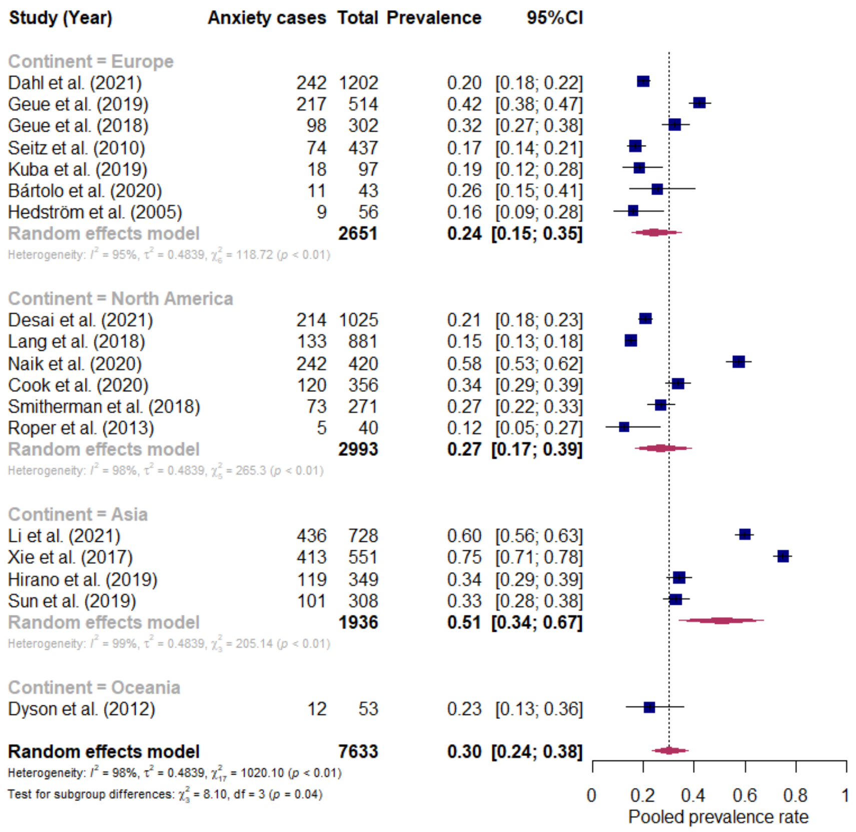

eFigure 23. Meta-analysis results on prevalence of anxiety among AYA cancer survivors by geographical region (excluding studies with unsatisfactory ratings)

Study (Year)	Anxiety cases	Total	Prevalence	95%CI
**Treatment = Majority no**				
Geue et al. (2018)	98	302	0.32	[0.27; 0.38]
**Treatment = No**				
Dahl et al. (2021)	242	1202	0.20	[0.18; 0.22]
Desai et al. (2021)	214	1025	0.21	[0.18; 0.23]
Geue et al. (2019)	217	514	0.42	[0.38; 0.47]
Lang et al. (2018)	133	881	0.15	[0.13; 0.18]
Naik et al. (2020)	242	420	0.58	[0.53; 0.62]
Seitz et al. (2010)	74	437	0.17	[0.14; 0.21]
Smitherman et al. (2018)	73	271	0.27	[0.22; 0.33]
Kuba et al. (2019)	18	97	0.19	[0.12; 0.28]
Roper et al. (2013)	5	40	0.12	[0.05; 0.27]
Random effects model		**4887**	**0.24**	**[0.16; 0.35]**
Heterogeneity: $I^2 = 98\%$, $\tau^2 = 0.5915$, $\chi^2_8 = 367.2$ ($p < 0.01$)				
**Treatment = Yes**				
Li et al. (2021)	436	728	0.60	[0.56; 0.63]
Xie et al. (2017)	413	551	0.75	[0.71; 0.78]
Hirano et al. (2019)	119	349	0.34	[0.29; 0.39]
Sun et al. (2019)	101	308	0.33	[0.28; 0.38]
Hedström et al. (2005)	9	56	0.16	[0.09; 0.28]
Random effects model		**1992**	**0.44**	**[0.28; 0.61]**
Heterogeneity: $I^2 = 98\%$, $\tau^2 = 0.5915$, $\chi^2_4 = 230.51$ ($p < 0.01$)				
**Treatment = Majority yes**				
Cook et al. (2020)	120	356	0.34	[0.29; 0.39]
Dyson et al. (2012)	12	53	0.23	[0.13; 0.36]
Bártolo et al. (2020)	11	43	0.26	[0.15; 0.41]
Random effects model		**452**	**0.27**	**[0.13; 0.49]**
Heterogeneity: $I^2 = 41\%$, $\tau^2 = 0.5915$, $\chi^2_2 = 3.37$ ($p = 0.19$)				
**Random effects model**		**7633**	**0.30**	**[0.23; 0.38]**
Heterogeneity: $I^2 = 98\%$, $\tau^2 = 0.5915$, $\chi^2_{17} = 1020.10$ ($p < 0.01$)				
Test for subgroup differences: $\chi^2_3 = 4.07$, df = 3 ($p = 0.25$)				

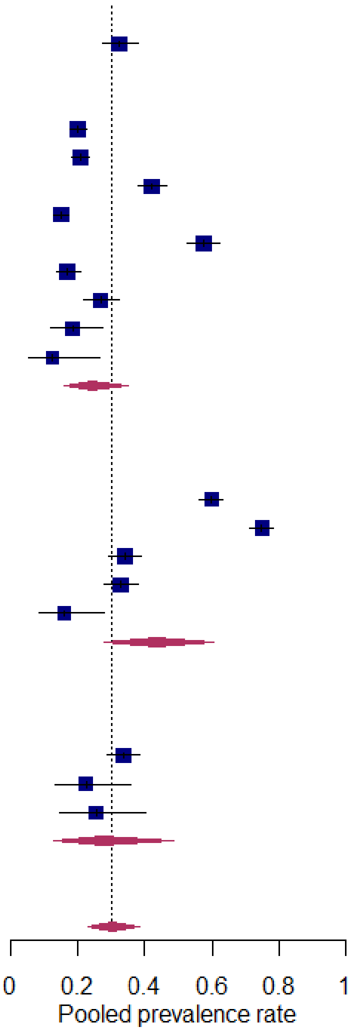

eFigure 24. Meta-analysis results on prevalence of anxiety among AYA cancer survivors by treatment status (excluding studies with unsatisfactory ratings)

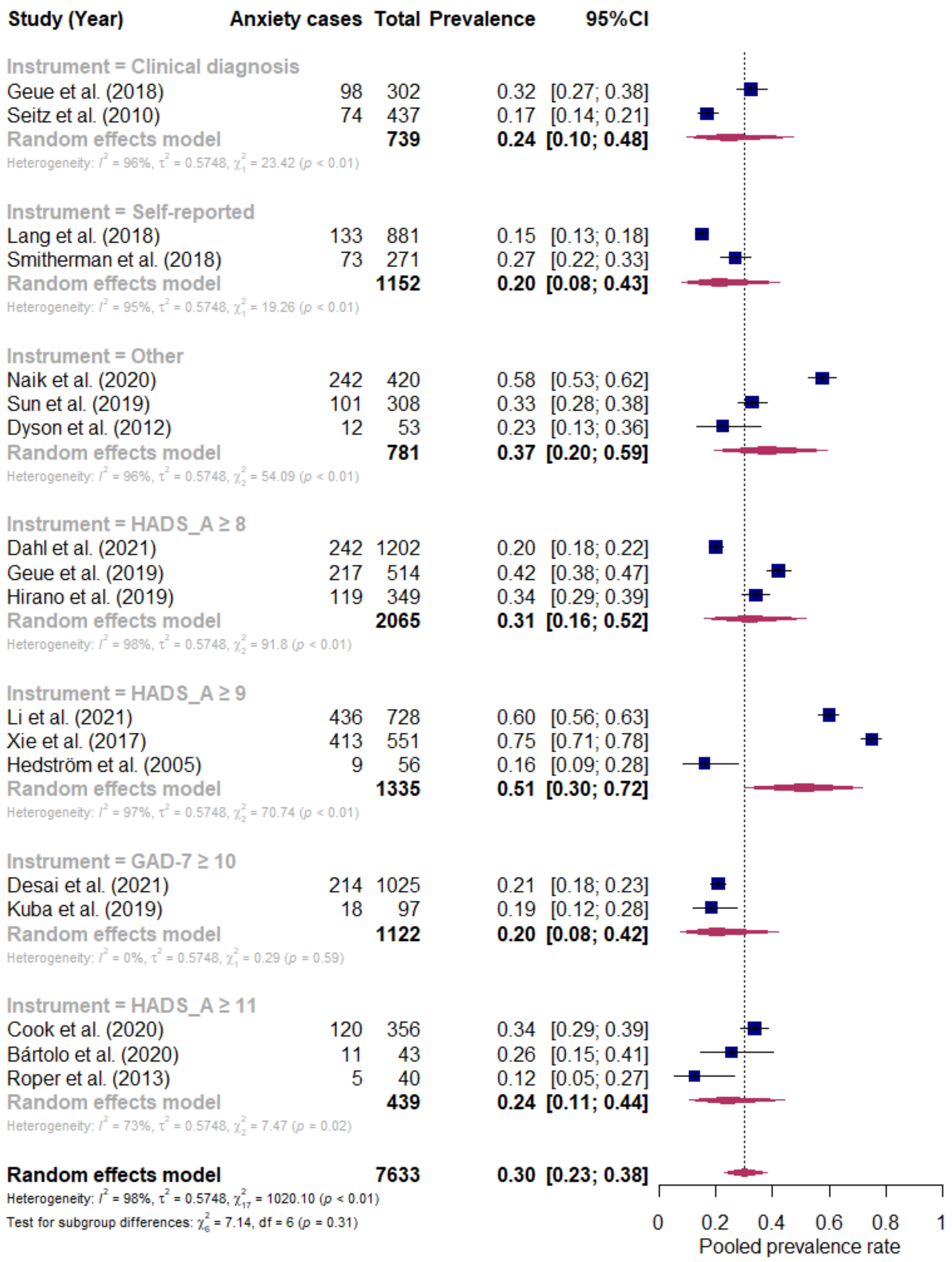

eFigure 25. Meta-analysis results on prevalence of anxiety among AYA cancer survivors by instrument (excluding studies with unsatisfactory ratings)

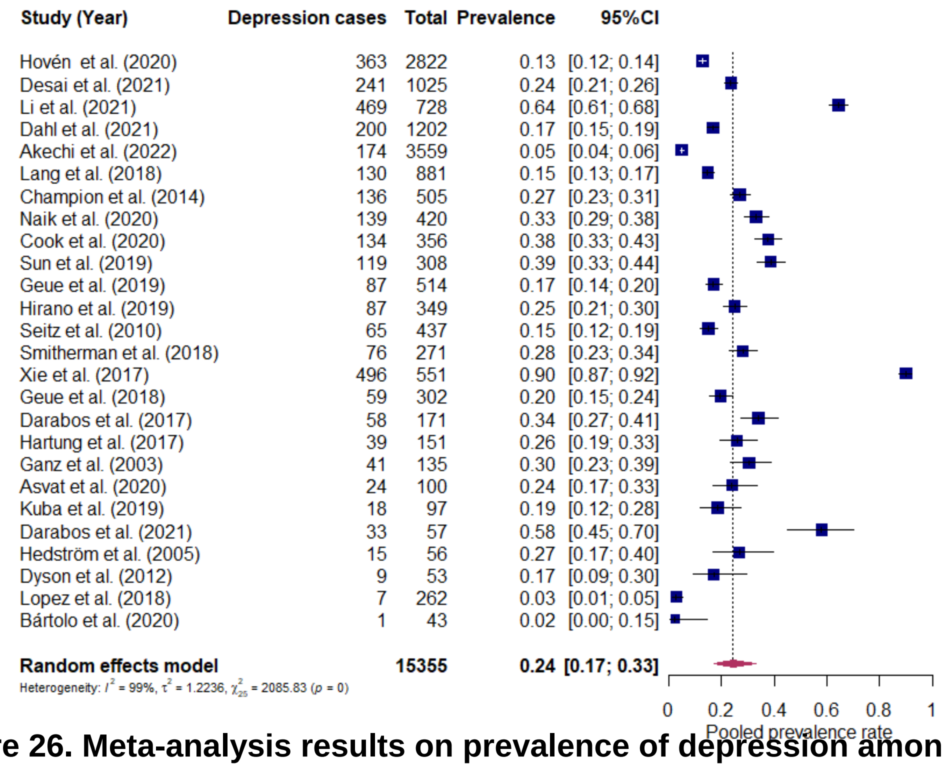

eFigure 26. Meta-analysis results on prevalence of depression among AYA cancer survivors (excluding studies with unsatisfactory ratings)

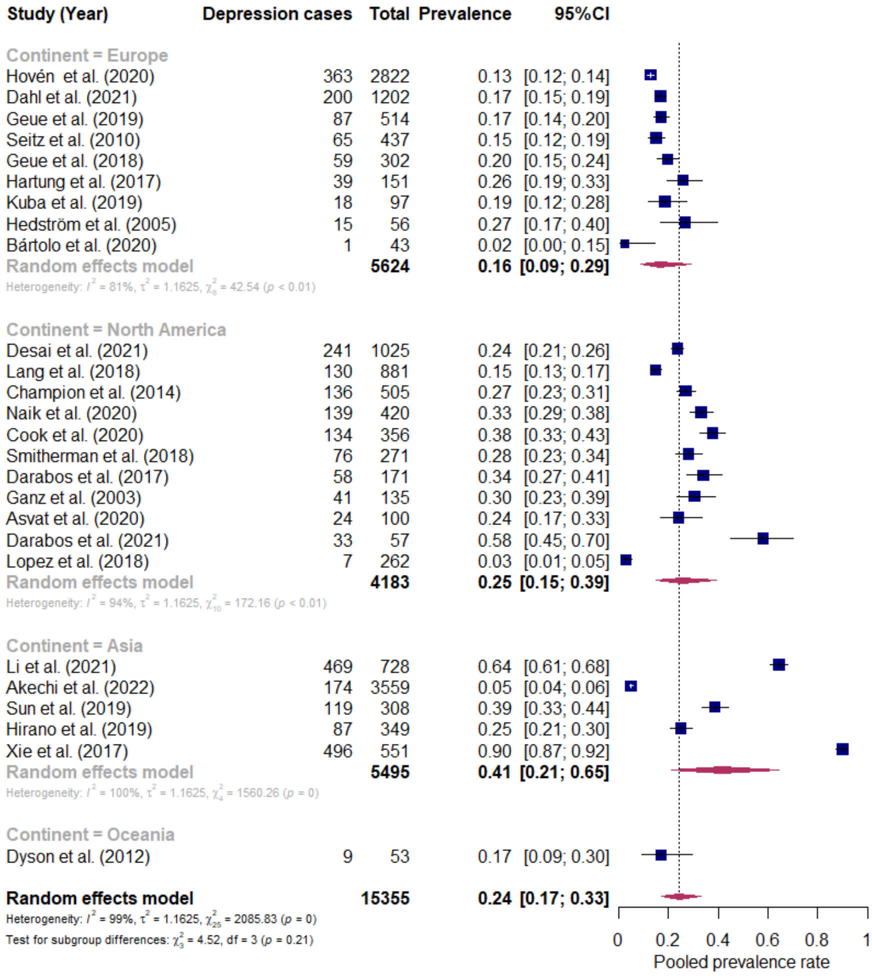

eFigure 27. Meta-analysis results on prevalence of depression among AYA cancer survivors by geographical region (excluding studies with unsatisfactory ratings)

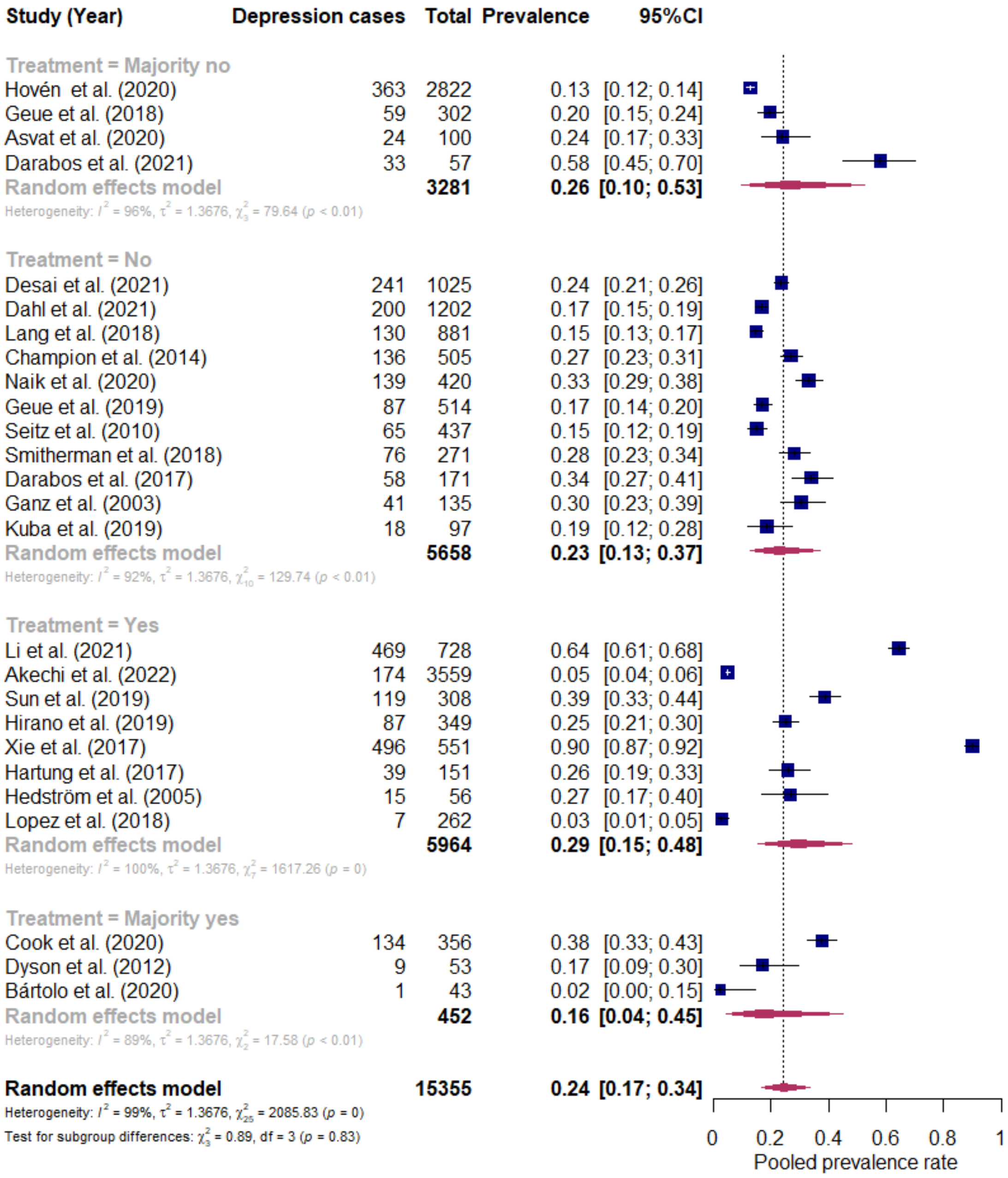

eFigure 28. Meta-analysis results on prevalence of depression among AYA cancer survivors by treatment status (excluding studies with unsatisfactory ratings)

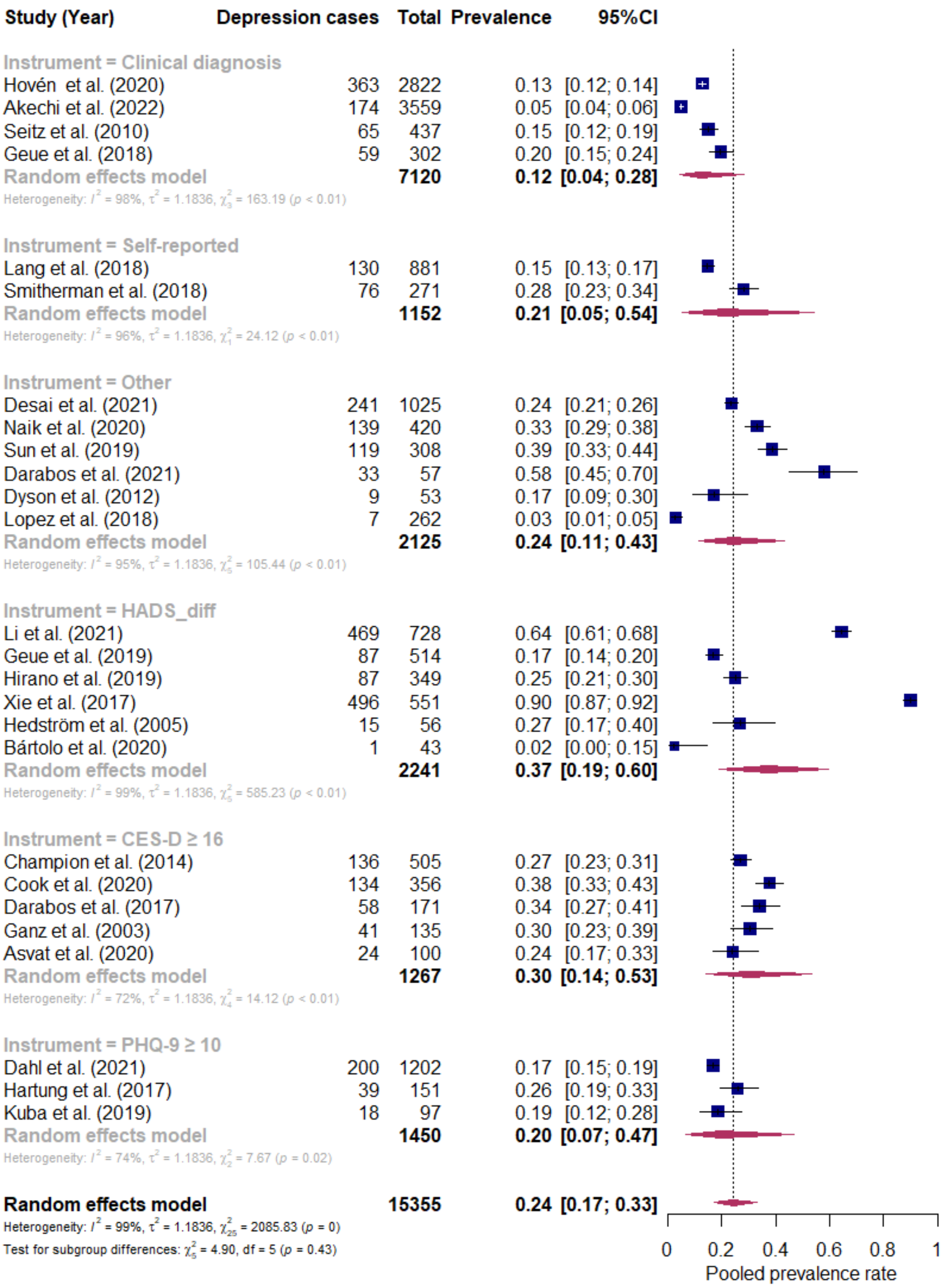

eFigure 29. Meta-analysis results on prevalence of depression among AYA cancer survivors by instrument (excluding studies with unsatisfactory ratings)

eTable 1. Eligibility criteria according to the PECOS scheme

PECOS components	Inclusion criteria	Exclusion criteria
**Population**	• Adolescent and young adult cancer survivors, from 15 to 39 years of age as the main study population<br>• Or the study included individuals within this age-group – e.g. as part of their stratified analysis<br>• Or the study population is defined as adolescent and young adult cancer survivors or adolescent survivors or young adult cancer survivors with a lower or higher age limit (+/-5 years from the NCI definition) at the moment of diagnosis, any type of cancer	• Childhood or adult cancer survivors not within the age range 15-39 and not defined as AYAs/adolescent cancers survivors/young adult cancer survivors (+/-5 years from the NCI definition)<br>• Only parents/siblings/partners of AYA cancer survivors<br>• Study defined population as AYAs, but the participants are diagnosed during childhood (starting from age 0)
**Exposure**	Cancer/ cancer treatment	-
**Comparator**	• No comparison group or comparing with normative values<br>• Siblings, healthy peers, other patients/survivors	-
**Outcome**	• Report of at least one of the outcomes: psychological distress, anxiety or depression in percentage (prevalence), risk estimates (RR, HR, OR) or mean differences/p-values (comparing with a healthy group or across different time points)<br>• Both self-reports and clinical diagnosis (based on ICD-10 reporting, DSM-4 and 5) of the outcomes before, during and after treatment for cancer<br>• Any anxiety disorder such as: generalized anxiety disorder (GAD), phobias, panic attacks or simply referred to as anxiety involving measuring of clinically significant symptoms such as "excessive anxiety, worry, fear, apprehension, and/or dread"*<br>• Depression referred to as mood disorder, major depressive disorder or depressive symptoms such as "affect, sleep disturbance, and thought patterns"*<br>• Psychological or emotional distress referring to a "multifactorial unpleasant experience of a psychological (i.e., cognitive, behavioral, emotional), social, spiritual, and/or physical nature that may interfere with one's ability to cope effectively with cancer"*, also identified as a summary measure of anxiety or depression	• No discussion (inclusion in the results) of the prevalence or risk measures of psychological distress, anxiety or depression<br>• Report of only distress in general without referral to psychological distress<br>• Prevalence of antidepressant medication only<br>• Only reports from parents/siblings/partners<br>• Only reports of sadness or acute episodes of anxiety<br>• Only other mental disorders<br>• Only discussing mental health in general<br>• Only mean values of the outcomes without comparing to another group or across time points
**Study design and language**	• Quantitative studies (cross-sectional, cohort and case-control) published in English up to May 2022	• Interventions<br>• Letter/report/comment/ case-reports-series/reviews/meta-analyses/abstracts/unpublished research<br>• Only qualitative study<br>• Study protocol/feasibility studies/concept analysis/validation studies

***Sources:** PDQ® Supportive and Palliative Care Editorial Board. PDQ Adjustment to Cancer. Bethesda, MD: National Cancer Institute. Updated <12.04.2023>. Available at: https://www.cancer.gov/about-cancer/coping/feelings/anxiety-distress-hp-pdq. Accessed <05.06.2023>. [PMID: 26389397]
PDQ® Supportive and Palliative Care Editorial Board. PDQ Depression. Bethesda, MD: National Cancer Institute. Updated <05.04.2023>. Available at: https://www.cancer.gov/about-cancer/coping/feelings/depression-hp-pdq. Accessed <05.06.2023>. [PMID: 26389407]

eTable 2. Search strategy used to identify eligible studies

Nr.	Search string
1	Depression OR depressive symptoms OR major depression OR clinical depression OR mood disorder OR emotional disturbances
	**OR**
2	Anxiety OR anxiety disorders OR nervousness OR generalized anxiety disorder OR phobia OR panic disorder OR panic attack OR social phobia
	**OR**
3	Psychological distress OR mental distress OR emotional distress OR distress OR psychological adjustment
	**OR**
4	Psychology OR psychological outcomes OR psychiatric outcomes OR emotional disturbances OR mental health OR mental disorders OR psychopathology
5	**1 OR 2 OR 3 OR 4**
6	Adolescent OR young adult OR emerging adults OR teen OR adolescent and young adults OR AYA
	AND
7	cancer OR tumor OR neoplasm OR neoplasms OR carcinoma OR neoplasia OR tumour OR oncology
	AND
8	survivors OR survivorship OR survivor OR long-term survivor
9	**6 AND 7 AND 8**
**FINAL_RESULT**	**#5 AND #9**

eTable 3. Selected characteristics of the included studies and quality assessment results (n= 68)

Author (Year)	Country	Study design	Number of AYA cancer survivors	Source of recruitment	Age at diagnosis	Current age	Type of cancer	Treatment status	Women (%)	Reported outcomes of interest	NOS rating (Max. 10 stars)
**Abdelhadi et al. (2022)**	USA	Cross-sectional	1757	Population-based survey	15-39	18+	All besides nonmelanoma skin cancers	Majority off treatment*	80	Distress	Good (7 stars)
**Ahomäki et al. (2015)**	Finland	Longitudinal	9543	Cancer registry	20-34	-	All	Off treatment	59	Anxiety and depression	Very good (9 stars)
**Akechi et al. (2022)**	Japan	Longitudinal	3559	Medical insurance /claims database	15-39	15-40	All	On treatment*	60	Depression	Very good (10 stars)
**Allen et al. (1997)**	UK	Cross-sectional	47	Hospital setting	12-20	-	All	On treatment	35	Anxiety and depression	Unsatisfactory (4 stars)
**Ander et al. (2016)**	Sweden	Longitudinal	61	Clinical setting	13-19	-	All	On treatment	39	Anxiety and depression	Unsatisfactory (4 stars)
**Asvat et al. (2020)**	USA	Cross-sectional	100	Clinical setting	15-39	18+	Primarily hematological cancer	Majority off treatment	40	Depression	Satisfactory (6 stars)
**Bártolo et al. (2020)**	Portugal	Cross-sectional	43	Clinical setting	18-40	26-40	Breast cancer	Majority on treatment	100	Anxiety and depression	Satisfactory (5 stars)
**Bitsko et al. (2008)**	USA	Cross-sectional	47	Unclear	10-21	-	The majority blood cancers	Off treatment	48	Depression	Unsatisfactory (2 stars)
**Adjei Boakye et al. (2022)**	USA	Cross-sectional	2671	General population National Health Interview Survey (NHIS)	15-39	Mean (SD) 52.8 (19.1)	All excluding nonmelanoma skin or other unkown	Off treatment*	-	Distress	Good (7 stars)

							skin cancer				
**Carter et al. (2010)**	USA	Longitudinal	71	Clinical setting	18-45	20-45	Cervical cancer	On treatment	100	Depression	Unsatisfactory (4 stars)
**Champion et al. (2014)**	USA	Cross-sectional	505	Database of trials	23-45	28-54	Breast cancer	Off treatment	100	Anxiety and depression	Satisfactory (6 stars)
**Chan et al. (2018)**	Singapore	Longitudinal	65	Clinical setting	15-39	-	Lymphoma, sarcoma, primary brain malignancies and germ cell tumors	Majority off treatment	45	Distress	Unsatisfactory (4 stars)
**Chen et al. (2020)**	USA	Longitudinal	179	Multiple sources: Hospital and university institutions	15-39	-	Any invasive cancer	Majority on treatment	47	Distress	Satisfactory (6 stars)
**Cook et al. (2020)**	USA	Longitudinal	356	Academic and community cancer centers recruited for an RCT study	18-45	18-45	Breast Cancer	Majority on treatment	100	All three outcomes	Satisfactory (5 stars)
**Dahl et al. (2021)**	Norway	Cross-sectional	1202	Cancer registry	19-39	24-64	All common AYA cancer	Off treatment*	74	Anxiety and depression	Good (8 stars)
**Darabos et al. (2017)**	USA	Cross-sectional	171	Cancer care registry	-	18-29	Testicular	Off treatment	0	Depression	Satisfactory (5 stars)
**Darabos et al. (2021)**	USA	Cross-sectional	57	Social media and cancer registry	18-39	24-42	All common AYA cancer	Majority off treatment	96	Depression	Satisfactory (6 stars)
**Desai et al. (2021)**	USA	Cross-sectional	1025	Multiple sources: Registries,	15-35	18-40	All common	Off treatment	100	Anxiety and depression	Satisfactory (6 stars)

				university, organizations, physicians			AYA cancer				
**Dewar et al. (2021)**	USA	Cross-sectional	2646	National Health Interview Survey	15-39	18+	All besides non-melanoma skin cancer and unspecified or unknown cancer types	Off treatment	82	Distress	Good (7 stars)
**Duan et al. (2021)**	China	Cross-sectional	809	Hospital setting	15-39	15-39	All common AYA cancers	Majority on treatment*	60	Distress	Satisfactory (6 stars)
**Dyson et al. (2012)**	Australia	Cross-sectional	53	Clinical setting	16-30	M (SD)<br><br>20.96 (0.46)	All	Majority on treatment	43	All three outcomes	Good (7 stars)
**Ganz et al. (2003)**	USA	Cross-sectional	135	Cancer center registry and hospital	25-39	30-50	Breast cancer	Off treatment	100	Depression	Satisfactory (5 stars)
**Garvey et al. (2018)**	Australia	Cross-sectional	29	Hospital setting	17-39	18-39	All	On treatment	-	Distress	Satisfactory (6 stars)
**Geue et al. (2014)**	Germany	Cross-sectional	149	Clinical setting + through press	-	18-45	All	Majority off treatment	66	Distress	Satisfactory (6 stars)
**Geue et al. (2018)**	Germany	Cross-sectional	302	Clinical setting	15-39	M (SD)<br><br>33.8 (6.5)	All	Majority off treatment	65	All three outcomes	Very good (9 stars)
**Geue et al. (2019)**	Germany	Longitudinal	514	Clinical setting and cancer registry	18-39	-	All	Off treatment	75	All three outcomes	Satisfactory (6 stars)

**Gunn et al. (2015)**	Finland	Longitudinal	315	Cancer registry	16-24	Median age<br><br>35.7	Brain tumors	Off treatment	46	Depression	Good (8 stars)
**Hall et al. (2016)**	Australia	Cross-sectional	145	Cancer registry	15-39	-	Hematological	Majority off treatment	-	Anxiety and depression	Satisfactory (6 stars)
**Hamilton et al. (2022)**	Canada	Cross-sectional	36	Clinical setting	18-39	Median<br><br>54 years	Head and neck cancer	Off treatment	61	Anxiety and Depression	Unsatisfactory (3 stars)
**Hartung et al. (2017)**	Germany	Cross-sectional	151	Clinical setting	-	18-35	All	On treatment	-	Depression	Good (7 stars)
**Hedström et al. (2005)**	Sweden	Cross-sectional	56	Clinical setting	-	13-19	All	On treatment	43	Anxiety and depression	Satisfactory (5 stars)
**Hirano et al. (2019)**	Japan	Cross-sectional	349	Clinical setting	15-39	15-39	All	On treatment	70	Anxiety and depression	Satisfactory (5 stars)
**Hovén et al. (2020)**	Sweden	Longitudinal	2822	Cancer registry	13-19	Median<br><br>18 years	All common AYA cancers	Majority off treatment*	51	Depression	Very good (9 stars)
**Hughes et al. (2015)**	Australia	Case-control (Cross-sectional analyses)	33	Clinical setting	15-25	-	All	On treatment	64	Anxiety and depression	Unsatisfactory (3 stars)
**Husson et al. (2020)**	Netherlands	Cross-sectional	84	Cancer registry	18-39	M (SD)<br><br>40.8 (7.6)	Thyroid cancer	Off treatment*	82	Distress	Satisfactory (6 stars)
**Jörngården et al. (2007)**	Sweden	Longitudinal	56	Clinical setting	13-19	-	All	Majority on treatment	43	Anxiety and depression	Good (7 stars)
**Kaul et al. (2017)**	USA	Cross-sectional	875	Population-based survey	15-39	20-85	All besides non-melanom	Off treatment*	77	Distress	Satisfactory (6 stars)

							a skin cancer				
**Kim et al. (2021)**	South Korea	Cross-sectional	32	Hospital setting	≤39	≤39	Breast cancer	On treatment	100	Anxiety and depression	Good (7 stars)
**Kuba et al. (2019)**	Germany	Cross-sectional	97	Cancer registry	18-45	-	Hematological	Off treatment	-	Anxiety and depression	Satisfactory (5 stars)
**Kwak et al. (2013)**	USA	Longitudinal	215	Clinical setting	14-39	-	All	On treatment at baseline	47	All three outcomes	Satisfactory (6 stars)
**Lane et al. (2021)**	Canada	Cross-sectional	461	Support and advocacy organization and media	15-39	20-39	All	Majority off treatment	87	Distress	Good (7 stars)
**Lang et al. (2018)**	Canada	Cross-sectional annual cycles	881	Population weighting survey	15-39	-	All	Off treatment	69	Anxiety and depression	Satisfactory (5 stars)
**Li et al. (2021)**	China	Cross-sectional	728	Hospital setting	15-39	15-40	All common AYA cancers	On treatment	59	All three outcomes	Satisfactory (5 stars)
**Lopez et al. (2018)**	USA	Cross-sectional	286	Clinical setting	15-39	-	All	On treatment	73	Anxiety and depression	Satisfactory (5 stars)
**McCarthy et al. (2016)**	Australia	Cross-sectional	196	Hospital setting	15-25	-	All	Majority on treatment*	49	Distress	Satisfactory (5 stars)
**Michel et al. (2019)**	Switzerland	Cross-sectional	160	Cancer registry	16-25	20-40+	Most cancer sites	Off treatment*	39	All three outcomes	Good (7 stars)
**Monteiro et al. (2013)**	Portugal	Cross-sectional	36	University setting	15-39	20-38	All	Majority off treatment	67	Anxiety and depression	Unsatisfactory (4 stars)
**Muffly et al. (2016)**	USA	Cross-sectional	61	Clinical setting	15-39	18-42	Hematological cancers	Majority off treatment	36	Anxiety and depression	Unsatisfactory (4 stars)
**Naik et al. (2020)**	Canada	Cross-sectional	420	Clinical setting	18-39	-	Breast cancer	Off treatment	100	Anxiety and depression	Good (7 stars)
**Okamura et al. (2022)**	Japan	Cross-sectional	206	Population	16-39	22-39	All common	Majority on treatment	87	Distress	Unsatisfactory

				web-based survey			AYA cancers				(4 stars)
**Olweny et al. (1993)**	Australia	Cross-sectional	102	Cancer registry and clinical setting	15-40	M<br><br>38.6	Hemato-logical cancers, testis, choriocar-cinoma, bone sarcoma and small cell cancer of the lung	Off treatment	42	Anxiety	Satisfactory<br>(6 stars)
**Patterson et al. (2021)**	Australia, Canada, UK, USA	Cross-sectional	288	Cancer centers	15-29	M (SD)<br><br>21.5 (3.8)	All	Majority on treatment	45	Distress	Satisfactory<br>(6 stars)
**Phillips-Salimi et al. (2013)**	USA	Case-control<br><br>(Cross-sectional analyses)	100	Population-based survey	15-29	M (SD)<br><br>39.5 (10.8)	Breast and gynecological cancers	Off treatment*	100	Distress	Good<br>(8 stars)
**Prasad et al. (2015)**	USA	Cross-sectional	2589	Clinical setting	11-21	25-35+	Hemato-logical, CNS malignan-cies, malignant kidney tumor, neuro-blastoma, soft tissue sarcoma, and malignant bone tumor	Off treatment*	50	Anxiety and depression	Good<br>(7 stars)

**Raphael et al. (2020)**	New Zealand	Cross-sectional	30	Cancer registry	-	18-39	Hematological cancer	Off treatment	41	Distress	Satisfactory (6 stars)
**Roper et al. (2013)**	USA	Longitudinal	40	Clinical setting	21-40	M (SD) 30.4 (5.7)	Hodgkin lymphoma	On treatment at baseline	61	All three outcomes	Satisfactory (5 stars)
**Salsman et al. (2014)**	USA	Cross-sectional	335	General population	18-39	M (SD) 31.8 (5.4)	All	Off treatment	68	Anxiety and depression	Satisfactory (5 stars)
**Seitz et al. (2010)**	Germany	Cross-sectional	820	Cancer registry	15-18	M (SD) 30.7 (5.9)	All	Off treatment	56	All three outcomes	Satisfactory (6 stars)
**Smitherman et al. (2018)**	USA	Cross-sectional	271	Clinical setting	15-39	30-50	Solid tumor majority	Off treatment*	74	Anxiety and depression	Satisfactory (5 stars)
**Soleimani et al. (2021)**	Canada	Cross-sectional	227	Cancer care center	18-39	Median age 28	Germ cell tumors	Majority on treatment*	0	Anxiety and depression	Unsatisfactory (4 stars)
**Sun et al. (2019)**	China	Cross-sectional	308	Clinical setting	15-39	-	Mostly breast cancer	On treatment	68	Anxiety and depression	Good (7 stars)
**Tan et al. (2020)**	Singapore	Longitudinal	91	Clinical setting	15-39	16-39	All except primary and secondary CNS tumors	On treatment	42	Distress	Unsatisfactory (4 stars)
**Tsuchiya et al. (2020)**	Japan	Cross-sectional	35	General population via market research company	-	20-40	All	Majority on treatment*	-	Distress	Unsatisfactory (4 stars)
**Vazquez et al. (2020)**	USA	Longitudinal	700	Academic and community	17-40	Age group (%)	Breast cancer	Majority on-treatment	100	Anxiety and depression	Unsatisfactory (4 stars)

				healthcare centers		<35 (33.3%) 35+ (66,7%)					
**Xie et al. (2017)**	China	Cross-sectional	551	Clinical setting	15-39	15-39	All	On treatment	60	All three outcomes	Satisfactory (6 stars)
**Yan et al. (2022)**	Canada	Cross-sectional	384	Social media platforms of cancer support groups and cancer clinics	15-39	18-49	All common AYA cancers	Off treatment	43	Distress	Good (7 stars)
**Zabora et al. (2001)**	USA	Cross-sectional	692	Clinical database	-	20-39	All	Majority on treatment*	-	Distress	Unsatisfactory (4 stars)
**Zebrack et al. (2014)**	USA	Longitudinal	152	Clinical setting	15-39	-	All	On treatment at baseline	45	Distress	Good (7 stars)

*A judgment was made based on time since diagnosis - when the majority of the population were long-term cancer survivors (more than 3 years from diagnosis), they were considered as majority off-treatment. If the majority of the population were inpatients, these were considered a majority on treatment cancer survivors.

eTable 4. Newcastle-Ottawa quality assessment scoring for the 68 included studies

Cross-sectional studies					
	Selection (Max. 5 stars)	Comparability (Max. 2 stars)	Outcome (Max. 3 stars)	Total (Max. 10 stars)	Rating
**Geue et al. (2018)**	****	**	***	9	Very good
**Lang et al. (2018)**	**	**	*	5	Satisfactory
**Michel et al. (2019)**	***	**	**	7	Good
**Muffly et al. (2016)**	**	-	**	4	Unsatisfactory
**Sun et al. (2019)**	***	**	**	7	Good
**Dyson et al. (2011)**	***	**	**	7	Good
**Hirano et al. (2019)**	***	-	**	5	Satisfactory
**McCarthy et al. (2016)**	***	-	**	5	Satisfactory
**Seitz et al. (2010)**	***	*	**	6	Satisfactory
**Hughes et al. (2015)**	**	-	*	3	Unsatisfactory
**Smitherman et al. (2018)**	***	*	*	5	Satisfactory
**Xie et al. (2017)**	***	*	**	6	Satisfactory
**Darabos et al. (2017)**	***	*	*	5	Satisfactory
**Garvey et al. (2018)**	**	**	**	6	Satisfactory
**Hall et al. (2016)**	****	-	**	6	Satisfactory
**Hartung et al. (2017)**	****	-	**	6	Satisfactory
**Allen et al. (1997)**	**	*	*	4	Unsatisfactory
**Geue et al. (2014)**	***	*	**	6	Satisfactory
**Olweny et al. (1993)**	****	*	*	6	Satisfactory

**Kuba et al. (2019)**	***	-	**	5	Satisfactory
**Salsman et al. (2014)**	**	*	**	5	Satisfactory
**Lopez et al. (2018)**	**	*	**	5	Satisfactory
**Prasad et al. (2015)**	***	**	**	7	Good
**Monteiro et al. (2013)**	*	*	**	4	Unsatisfactory
**Bitsko et al. (2008)**	-	*	*	2	Unsatisfactory
**Champion et al. (2014)**	**	**	**	6	Satisfactory
**Hedström et al. (2005)**	**	*	**	5	Satisfactory
**Ganz et al. (2003)**	**	*	**	5	Satisfactory
**Zabora et al. (2001)**	**	*	*	4	Unsatisfactory
**Phillips-Salimi et al. (2013)**	***	**	***	8	Good
**Kaul et al. (2016)**	**	**	**	6	Satisfactory
**Asvat et al. (2020)**	**	**	**	6	Satisfactory
**Lane et al. (2019)**	***	**	**	7	Good
**Naik et al. (2020)**	****	*	**	7	Good
**Abdelhadi et al. (2022)**	***	**	**	7	Good
**Bartolo et al. (2020)**	**	*	**	5	Satisfactory
**Adjei Boakye et al. (2022)**	***	**	**	7	Good
**Dahl et al. (2021)**	****	**	**	8	Good
**Darabos et al. (2021)**	***	**	*	6	Satisfactory
**Desai et al. (2021)**	**	**	**	6	Satisfactory

**Dewar et al. (2021)**	***	**	**	7	Good
**Duan et al. (2021)**	***	*	**	6	Satisfactory
**Hamilton et al. (2022)**	**	-	*	3	Unsatisfactory
**Husson et al. (2020)**	****	*	*	6	Satisfactory
**Kim et al. (2021)**	****	**	*	7	Good
**Li et al. (2021)**	***	*	*	5	Satisfactory
**Raphael et al. (2020)**	***	*	**	6	Satisfactory
**Okamura et al. (2021)**	-	**	**	4	Unsatisfactory
**Patterson et al. (2021)**	***	*	**	6	Satisfactory
**Soleimani et al. (2021)**	**	*	*	4	Unsatisfactory
**Tsuchiya et al. (2020)**	*	**	*	4	Unsatisfactory
**Vazquez et al. (2020)**	***	-	*	4	Unsatisfactory
**Yan et al. (2022)**	***	**	**	7	Good
			**Longitudinal**		
	**Selection (Max. 5 stars)**	**Comparability (Max. 2 stars)**	**Outcome (Max. 3 stars)**	**Total (Max. 10 stars)**	**Rating**
**Ahomäki et al. (2015)**	****	**	***	9	Very good
**Akechi et al. (2022)**	*****	**	***	10	Very good
**Zebrack et al. (2014)**	***	**	**	7	Good
**Kwak et al. (2013)**	***	-	***	6	Satisfactory
**Jörngården et al. (2007)**	***	**	**	7	Good
**Ander et al. (2016)**	**	-	**	4	Unsatisfactory
**Chan et al. (2018)**	**	-	**	4	Unsatisfactory

**Roper et al. (2013)**	**	-	***	5	Satisfactory
**Carter et al. (2010)**	**	-	**	4	Unsatisfactory
**Gunn et al. (2015)**	****	*	***	8	Good
**Chen et al. (2020)**	**	*	***	6	Satisfactory
**Geue et al. (2019)**	**	*	***	6	Satisfactory
**Cook et al. (2020)**	**	-	***	5	Satisfactory
**Hovén et al. (2020)**	****	**	***	9	Very good
**Tan et al. (2020)**	**	-	**	4	Unsatisfactory

eTable 5. Main findings on prevalence of psychological distress, anxiety and depression in AYA cancer survivors, comparison with reference groups and predictors

Study (Year)	Number of AYA cancer survivors/ comparison group	Time of assessment	Assessment tool and cut-off	Prevalence (%)	Differences in AYA cancer survivors vs. comparison groups	Findings on predictors
**Outcome: Psychological distress**						
**Abdelhadi et al. (2022)**	1757/5227 cancer-free adults	0-20+ years since diagnosis	K6 ≥ 13	11.5	-	Female, lower income, low physical activity, unmarried, public insurance, smoking - Distress ↑<br>No differences in prevalence based on time since diagnosis
**Adjei Boakye et al. (2022)**	2671/2500 middle/older adult survivors and 1609 cancer-free adults (general population)	NA	K6 ≥ 13	7.0[a]	No differences for distress compared to general population, but higher risk of distress in AYAs than older survivors	Female – moderate distress ↑<br>Unmarried, uninsured, visiting a mental health professional - severe distress ↑<br>≥2 comorbid conditions, lower educational attainment – moderate and severe ↑
**Cook et al. (2020)**	356	Newly diagnosed	PSS ≥27	6.7[b]	**-**	**-**
**Dewar et al. (2021)**	2646/228029 cancer-free adults from the general population	10+ years since diagnosis	K6 ≥ 13	7.6[c]	Psychological distress in cancer survivors ↑	Diagnosis as young adult, brain cancer, cognitive dysfunction – Distress ↑<br>Being married – distress ↓
**Dyson et al. (2012)**	53	Within 4 years since diagnosis	STAI-S 0.5 SD above	25.0	-	No gender differences

			normative values and BDI-FS ≥ 4			
**Duan et al. (2021)**	809	Majority 0-3 years	DT ≥ 4	83.4	-	Female, divorced, digestive system malignancies, breast cancer, head and neck cancer, chemotherapy/radiotherapy - distress↑
**Garvey et al. (2018)**	29	4-9 months after diagnosis	DT ≥ 4	41.4	-	-
**Geue et al. (2014)**	149	Less than 4 years (83.2%) More than 4 years since diagnosis (16.1%)	PHQ - 4<br>Moderate (6-8)<br>Severe (9 - 12)	 5.4<br>4.0	-	Female –Psychological distress ↑
**Geue et al. (2018)**	302	4 weeks	(CIDI-O) – at least one mental disorder according to ICD-10	46.7	-	-
		1 year		55.4		
		Lifetime prevalence		69.5		
**Husson et al. (2020)**	84	M (SD)<br>11.2 (5.4) years	HADS ≥ 11	13.8	Proportion of HADS casesness differed significantly between AYAs (13.8%), middle-aged (28.7%) and older adults (22.2%)	More negative illness perceptions – distress ↑
**Kaul et al. (2017)**	875/875 cancer-free peers	M (SD)<br>23 (13.9) years	K6 ≥ 13	8.4	Psychological distress ↑ in cancer patients	Not being able to afford mental health care, not having seen a mental health professional within the previous year, being 15-19, survivors with ≥ 2 comorbidites, public insurance or no insurance,

						current smoker – Psychological distress ↑
**Lane et al. (2021)**	461/461 cancer-free peers	Mean (SD)<br><br>3.78 (3.83) years	K10 ≥ 25	48.4[d]	Psychological distress ↑ in cancer patients	Being out of school and work,body image dissatisfaction, lack of social support, higher fear of cancer recurrence – distress ↑
**Li et al. (2021)**	728	Mjaority up to 3 years since diagnosis	DT≥4	84.9	-	-
**McCarthy et al. (2016)**	196	Within 6–24 months from diagnosis	K10 ≥ 22 (Moderate to severe)	31.0	-	-
**Michel et al. (2019)**	160/358 cancer-free adults	M (SD)<br><br>12.4 (4.8) years	BSI-18 T≥ 57 in at least 2 scales or GSI T≥ 57	21.3	No differences found between AYA and cancer-free adults	Migrational background-Psychological distress ↑
**Okamura et al. (2022)**	206	Majority within 5 years from diagnosis	K6 ≥5	55.3	Lower prevalence of distress at 1-4 years since diagnosis, higher in 1 year and more than 10 years since diagnosis	Within 1 year and 10 years of after diagnosis, pain, change in income, change in work/school life, poor social support – distress ↑
**Patterson et al. (2021)**	288	Within 3 months since diagnosis	DT ≥ 5	41.5	-	Female, older AYAs (20-29 vs. 15-19) – distress ↑
**Phillipps-Salimi et al. (2013)**	100/300	M (SD)<br><br>16.7 (10.0) years since diagnosis	K6 ≥ 13	19.4	-	-
**Raphael et al. (2020)**	30	Within 5 years since diagnosis	DT≥4	36.8	-	-
**Seitz et al. (2010)[e]**	437/516 cancer-free peers	M (SD)<br><br>13.68 (6.02) years since diagnosis	DIA-X/M-CIDI<br><br>At least one mental	24.3	Psychological distress ↑ in cancer patients	-

			disorder diagnosis			
**Xie et al. (2017)**	551	NR	DT ≥ 4	89.1	-	Gynecological, digestive system and breast tumors; chemotherapy or radiotherapy alone; 21-25 years old - Psychological distress ↑
**Yan et al. (2022)**	384	Majority within 5 years since diagnosis	K10 ≥25	66.9	-	Higher income and presence of pre-existing chronic physical health condition - distress ↑
**Zabora et al. (2001)**	692	Majority within 3 months after diagnosis	BSI<br>GSI<br>T≥ 63	38.3[f]	-	-
**Outcome: Anxiety**						
**Allen et al. (1997)**	47/173 age and sex-matched healthy peers	Median time from diagnosis 3 weeks	STAI	-	No differences found anxiety overall; Control group- more trait anxiety (p=0.017)	Female –Anxiety ↑
**Bártolo et al. (2020)**	43/37 controls	Mean<br>3 years since diagnosis	HADS ≥11	25.6	No differences in anxiety levels	-
**Champion et al. (2014)**	505/ 404 acquaintances; 622 older survivors	3-8 years post- diagnosis	STAI	-	State and trait anxiety ↑ than and older adults (p<.0001) but not ↑ than health-peers (p<.0.8873)	-
**Cook et al. (2020)**	356	Newly diagnosed	HADS ≥11	34.2[g]	-	No links between loss in household income and anxiety
**Dahl et al. (2021)**	1202/1690 younger cancer survivors and	Median (range)<br><br>14 (5-30) years	HADS-A ≥ 8	20.2	No differences in anxiety levels between YA and younger cancer survivors	-

	1453 cancer-free adults					
**Desai et al. (2021)**	1025	Majority 5+ years since diagnosis	GAD-7 ≥ 10	20.9[h]	-	Social support – anxiety ↓<br>Sexual minority in 19-39 years – Anxiety ↑
**Dyson et al. (2012)**	53	Within 4 years since diagnosis	STAI-S<br>0.5 SD above normative values	23.0	-	No sex differences
**Geue et al. (2018)**	302	Within 5 years since diagnosis	GAD-7 ≥9	20.8	-	Female - Anxiety ↑
		4 weeks	CIDI-O	24.4		
		1 year		32.3		
		Lifetime prevalence		44.5		
**Hamilton et al. (2022)**	36	Median<br>22 years	PSSCAN-R ≥ 8	14.0	-	-
**Hedström et al. (2005)**	56	4-8 weeks after diagnosis	HADS ≥ 9	16.0[i]	-	Female – Anxiety ↑
**Hirano et al. (2019)**	349	NR	HADS ≥ 8	34.0	-	-
**Hughes et al. (2015)**	33/99 older patients matched for sex and cancer type	NR	ESAS	43.0	No differences found	-
**Kim et al. (2021)**	32/94 older survivors	NA	HADS	-	Higher anxiety in AYA cancer survivors	-
**Kuba et al. (2019)**	97	From 2.5 to 26 years post-diagnosis	GAD-7 ≥ 10	19.0	-	-
**Lang et al. (2018)**	881/82889 cancer-free peers	NR	Self-reported health care professional	15.1	Anxiety ↑ in cancer survivors	-

			anxiety disorder			
**Li et al. (2021)**	728	Majority within 3 years from diagnosis	HADS ≥9	59.9	-	-
**Lopez et al. (2018)**	262/1879 older patients	NR	MYCaW	-	Anxiety ↑ in AYA cancer survivors	
**Michel et al. (2019)**	160/358 cancer-free adults	M (SD)<br><br>12.4 (4.8) years	BSI-18<br>T≥ 57	-	No differences found in anxiety levels	-
**Monteiro et al. (2013)**	36/435 cancer-free peers	M (SD)<br>1.73 (1.19) years	HADS ≥ 11	-	No differences in anxiety scores	-
**Muffly et al. (2016)**	61	13.2 (Median) months since diagnosis	STAI-S 0.5 SD above normative values	23.0	-	-
**Naik et al. (2020)**	420/10314 older survivors	Within 6 months of their diagnosis	PSSCAN-R ≥ 8	58.6	Anxiety in YAs compared to older patients ↑	Patients with metastatic disease and those living in smaller communities – symptoms of anxiety ↑
**Olweny et al. (1993)**	62/ 95 neighborhood controls and 78 cardiac patients	At least 5 years after diagnosis	HADS<br><br>Goldberg's Clinical Interview Schedule (CIS)	-	Anxiety ↑ than controls ($p=0.0083$) and ↓ than cardiac patients ($p= 0.047$)	No change in anxiety after controlling for sex
**Salsman et al. (2014)**	33/335 controls from the general population	Up to 5 years after diagnosis	MHI-18	-	No differences in anxiety levels (p-value 0.76)	25-to 29-years-old - Anxiety ↑ compared to those in the 30- to 39-year-old age group
**Seitz et al. (2010)[e]**	437/516 cancer-free peers	M(SD)<br>13.68 (6.02) years since diagnosis	DIA-X/M-CIDI<br><br>12-month prevalence	16.5	Anxiety ↑ compared to both cancer-free peers and normative scores	Female - Anxiety ↑

**Smitherman et al. (2018)**	271	Majority of individuals 1-5 years from diagnosis	Self-reported	27.0	-	-
**Soleimani et al. (2021)**	227/122 older cancer survivors	Median (range)<br><br>36 days (1-191 days)	PSSCAN-R	39.2	Subclinical and clinical anxiety higher in the AYAs (p=0.028)	-
**Sun et al. (2019)**	308	NR	GAD-7 ≥ 5	32.9	-	Being single, pessimistic, having more concurrent stressful life events and physical comorbidity - Anxiety ↑
**Xie et al. (2017)**	551	NR	HADS ≥ 9	75.0	-	21-25 years old - Anxiety ↑
**Vazquez et al. (2020)**	700	Median (range)<br><br>144 days (70-382 days)	HADS ≥ 11	22.6[j]	-	-
**Outcome: Depression**						
**Allen et al. (1997)**	47/173 age and sex-matched healthy peers	Median time from diagnosis 3 weeks	BDI 19-29 moderate to severe depression	5.0	No differences found on depression overall	Female – Depression ↑
**Akechi et al. (2022)**	3559/35590 age-matched healthy peers	12 months after diagnosis	ICD-10 code F32 (depressive episode), F33 (MDD, recurrent)	4.9	-	Males ≥25 years, multiple cancer categories , chemotherapy Depression ↑
**Asvat et al. (2020)**	100	Majority within 5 years of diagnosis	CES-D ≥16	24.0	-	Frequent tobacco and cannabis – Depression ↑
**Bártolo et al. (2020)**	43/37 controls	Mean<br>3 years since diagnosis	HADS ≥ 11	2.3	No differences in depression levels	-
**Bitsko et al. (2008)**	47	2-10 years post-diagnosis	CES-D ≥16	17.0[k]	-	Higher rates of happiness – Depression ↓

						Thinking negatively about one’s past- Depression ↑
**Champion et al. (2014)**	505/ 404 acquaintances; older survivors 622	3-8 years post- diagnosis	CES-D ≥ 16	27.0	Depression ↑ in AYA cancer survivors in comparison with both groups ($p<.0001$)	-
**Cook et al. (2020)**	356	Newly diagnosed	CES-D ≥16	37.6	-	No links between loss in household income and anxiety and depression
**Dahl et al. (2021)**	1202/1690 younger cancer survivors and 1453 cancer-free adults	Median (range)<br><br>14 (5-30) years	PHQ-9 ≥ 10	16.6	Higher prevalence rates of depression than cancer-free adults but no differences in comparison to younger survivor groups	-
**Darabos et al. (2017)**	171	M (SD)<br>31.30 (13.56) months since diagnosis	CES-D ≥ 16	34.0	-	Higher levels of social constraints and of restrictive emotionality – Depression ↑
**Darabos et al. (2021)**	57	Within 5 years since diagnosis	CES-D ≥10	56.3	-	-
**Desai et al. (2021)**	1025	Majority 5+ years since diagnosis	PHQ-8 ≥10	23.5	-	Social support – Depression ↓
**Dyson et al. (2012)**	53	Within 4 years since diagnosis	BDI-FS ≥ 4	17.0	-	No gender differences
**Ganz et al. (2003)**	135	M (SD)<br>6.35 (2.0)	CES-D ≥ 16	30.0[l]	No differences between younger survivors and older survivors (p-value =0.06)	-
**Geue et al. (2018)**	302	Within 5 years since diagnosis	PHQ-9 ≥9	36.1		
		4 weeks	CIDI-O	8.6		
		1 year		19.5		

		Lifetime prevalence		29.1		
**Hamilton et al. (2022)**	36	Median<br><br>22 years	PSSCAN-R ≥ 8	24.0	-	-
**Hartung et al. (2017)**	151	14 (Mean) months since diagnosis	PHQ-9 ≥ 10	26.0	-	-
**Hedström et al. (2005)**	56	4-8 weeks after diagnosis	HADS ≥ 7 (13-17)<br>HADS ≥ 8 (older)	26.0	-	Pain from procedures/treatments and weight changes higher in patients -Depression ↑
**Hovén et al. (2020)**	2822	Majority within 5 years since diagnosis	Swedish patient registry ICD-10 Mood disorders F3	12.8[m]	-	-
**Hirano et al. (2019)**	349	NR	HADS ≥ 8	25.0	-	-
**Hughes et al. (2015)**	33/99 older patients matched for sex and cancer type	NR	ESAS	48.0	No differences in depression levels	-
**Kim et al. (2021)**	32/94 older survivors	NA	HADS	-	No differences in depression levels	-
**Kuba et al. (2019)**	97	From 2.5 to 26 years post-diagnosis	PHQ-9 ≥ 10	19.0	-	-
**Lang et al. (2018)**	881/82889 cancer-free peers	NR	Self-reported health care professional mood disorder	14.8	Depression ↑ in cancer survivors	-
**Li et al. (2021)**	728	Majority within 3 years from diagnosis	HADS ≥9	64.4	-	-

**Lopez et al. (2018)**	262/1879 older patients	NR	MYCaW<br><br>ESAS (for comparisons)	2.7	No differences in depression scores	-
**Michel et al. (2019)**	160/358 cancer-free adults	M (SD)<br><br>12.4 (4.8) years	BSI-18<br>T≥ 57	-	No differences in depression scores	Not being in partnership - Depression ↑
**Monteiro et al. (2013)**	36/435 cancer-free peers	M (SD)<br>1.73 (1.19) years	HADS ≥ 11	-	No differences in depression scores	-
**Muffly et al. (2016)**	61	13.2 (Median) months since diagnosis	CES-D ≥16	28.0	-	-
**Naik et al. (2020)**	420/10314 older survivors	Within 6 months of their diagnosis	PSSCAN-R ≥ 8	33.6	Depression in YAs compared to older patients ↑	Patients with metastatic disease and those living in smaller communities – symptoms of depression ↑
**Salsman et al. (2014)**	33/335 controls from the general population	Up to 5 years after diagnosis	MHI-18	-	No differences found	25-to 29-years-old - Depression ↑ compared to those in the 30- to 39-year-old age group
**Seitz et al. (2010)[e]**	437/516 cancer-free peers	M(SD)<br>13.68 (6.02) years since diagnosis	DIA-X/M-CIDI<br><br>12-month prevalence	14.9	Depression ↑ compared to cancer-free peers (p=0.01)	Female - Depression ↑
**Smitherman et al. (2018)**	271	Majority of individuals 1-5 years from diagnosis	Self-reported	28.0	-	-
**Soleimani et al. (2021)**	227/122 older cancer survivors	Median (range)<br>36 days (1-191 days)	PSSCAN-R	26.5	No differences found	-
**Sun et al. (2019)**	308	NR	PHQ-9 ≥5	38.6	-	Being single, pessimistic, having more concurrent stressful life events and physical comorbidity - Depression ↑

**Vazquez et al. (2020)**	700	Median (range)<br>144 days (70-382 days)	HADS ≥ 11	7.1	-	-
**Xie et al. (2017)**	551	NR	HADS ≥ 9	90.0	-	21-25 years old - Depression ↑

Note: Only studies reporting on prevalence and no longitudinal changes were included in this table; The prevalence might be slightly different than in the forest plots, due to rounding. For each 'Assessment tool', the cut-off is presented; When there is no prevalence presented but only screening instrument – prevalence was not reported but only comparisons in means/medians between AYA and comparisons were made; ↑ Represents higher rates of the outcomes in cancer survivors based on the predictor/risk factor and ↓ represents lower rates;

[a]Estimated for both AYA groups included in the study; [b]Estimated for all income groups together; [c]Estimated for AYAs with and without cognitive disfunctions; [d]Estimated for those experiencing at least moderate distress; [e]Numbers based only on clinical interviews and the estimated prevalence was based on CIDI and comparisons were made through HADS scores; [f]Estimated only for those aged 20 to 39; [g]Estimated for all income groups; [h]Estimated for both groups (sexual minority + heterosexual); [i]Estimated for the cut-off 9; [j]Estimated for all participants (both with PTSD and without); [k]Estimated for mild, moderate and severe symptoms; [l]Estimated for all participants in AYA range; [m]Estimated from diagnosis of mood disorders for men and women overall;

M, Mean; SD, Standard Deviation; NR, Not Reported; MDD, Major depressive disorder; CIDI – O, Composite International Diagnostic Interview for Oncology; PHQ-4, Patient Health Questionnaire-4; PHQ-9, Patient Health Questionnaire-9; BSI -18, Brief Symptom Inventory – 18; GSI - Global Symptom Index; STAI-S, State-Trait Anxiety Inventory – State; BDI, Beck Depression Inventory; BDI-FS, Beck Depression Inventory 2nd edition—Fast Screen; K10, The Kessler Psychological Distress Scale – 10; DT, Distress Thermometer; K6, The Kessler Psychological Distress Scale – 6; HADS, The Hospital Anxiety and Depression Scale; CES-D, Center for Epidemiologic Studies Depression Scale; GAD-7, Generalized Anxiety Disorder Scale; DIA-X/M-CIDI, Expert System for Diagnosing Mental Disorders; ESAS, Edmonton Symptom Assessment Scale; MYCaW, Measure yourself concerns and wellbeing; PSS, Perceived Stress Scale; PSSCAN-R, The Psychosocial Screen for Cancer

eTable 6. Main findings on risk of developing psychological distress, anxiety and depression in AYA cancer survivors (n=16)

Study (Year)	Number of cancer survivors/controls	Instrument, definition and risk estimate (95% CI) for psychological distress	Instrument, definition and risk estimate (95% CI) for anxiety	Instrument and definition and risk estimate (95% CI) for depression	Adjusted for	Findings from subgroup analysis
**Ahomäki et al. (2015)**	9543/43,392 siblings[a]	-	Linkage to hospital discharge registry Based on ICD10 reporting<br><br>**HR, 1.2 (1.0–1.5)**	Linkage to hospital discharge registry Based on ICD10 reporting<br><br>**HR, 1.3 (1.1–1.5)**	Treatment era	Survivors of CNS tumors, soft tissue sarcomas and non-Hodgkin lymphomas (NHL) Depression ↑<br><br>Female, CNS tumors, ALL and HL – Anxiety ↑
**Lang et al. (2018)**	881/82889 cancer-free peers	-	Self-reported health care professional diagnosis<br><br>**OR, 2.2 (1.44-2.77)**	Self-reported health care diagnosis<br><br>**OR, 2.2 (1.7-2.86)**	Sex, physical comorbidities[b]	-
	881/13711 older cancer patients		**OR, 2.15 (1.60-2.89)**	**OR, 2.04 (1.45-2.86)**	Only physical comorbidities	
**Phillips-Salimi et al. (2013)**	100/300 Breast and gynecological cancers	K6 ≥ 13<br><br>**OR, 4.23 (2.08-8.62)**	-	-	Education; age and sex-matched controls	-
**Seitz et al. (2010)**	820/1027[c] cancer free peers	-	HADS ≥ 11<br><br>**OR, 1.65 (1.16–2.12)**	HADS ≥ 11<br><br>**OR, 1.69 (1.01–2.81)**	Sex, age, education	-

	437/516[d]<br>cancer free peers		DIA-X/M-CIDI<br><br>**OR, 1.59 (1.09 – 2.31)**	DIA-X/M-CIDI<br><br>**OR, 1.7 (1.15 – 2.53)**		
**Hall et al. (2016)**	145/175 older patients	-	DASS-21 ≥ 8<br><br>**OR, 1.48 (0.58, 3.76)**	DASS-21 ≥ 10<br><br>**OR, 1.29 (0.51-3.25)**	Unclear	-
**Kuba et al. (2019)**	96/96 cancer-free peers	-	GAD-7 ≥10<br><br>**RR, 3 (1.2-7.2)**	PHQ-9 ≥ 10<br><br>**RR, 18 (2.5 – 132.2)**	Unclear	-
**Prasad et al. (2015)**	2,589/390 siblings	-	BSI – 18<br>T ≥ 63<br><br>**OR, 2 (1.17 – 3.43)**	BSI – 18<br>T ≥ 63<br><br>**OR, 1.55 (1.04- 2.30)[e]**	Current age and sex	-
**Naik et al. (2020)**	420/10314 older survivors		PSSCAN-R ≥ 8<br><br>**OR, 2.49 (2.00–3.09)**	PSSCAN-R ≥ 8<br><br>**OR, 1.47 (1.17–1.84)**	Gender, stage, community size and income	-
**Gunn et al. (2015)**	315/2207 siblings	-	-	Registry linkage<br><br>**HR, 1.7 (0.8–3.4)**	Sex and birth cohort	**-**
**Abdelhadi et al. (2022)**	1757/5227 no history of cancer	K6 ≥ 13<br><br>**OR, 1.78 (1.37-2.40)**	-	-	Chronic conditions, age at survey, sex, race/ethnicity, education, marital status, income, insurance, exercise, BMI, smoking status	-
**Akechi et al. (2022)**	3559/35590 age-matched cancer free	-		ICD-10 code F32 (depressive episode) or F33 (MDD, recurrent)<br><br>**HR, 3.12 (2.64-3.70)**	Sex and working status	Leukemia, stomach cancer, brain/central nervous system cancer, lung cancer, colorectal cancer, male genitalia cancer, malignant lymphoma<br>Depression ↑

**Dahl et al. (2021)**	1202/1690 younger cancer survivors	-	-	PHQ-9 ≥ 10<br><br>**OR, 0.89 (0.51-1.57)**	Sex and age at survey	Female only on PHQ-9 definition – Depression ↑
**Dewar et al. (2021)**	2646/228029 cancer-free adults from the general population	K6<br>None/Low: 0-5<br>Moderate: 5-13<br>Severe: 13-24<br><br>**AOR, 1.60 (1.41-2.83)**	-	-	Age, sex, annual family income, race, ethnicity, marital status, sexual orientation, insurance status, and highest education level	Diagnosis as young adult, brain cancer, cognitive dysfunction – Distress ↑<br>Married – distress ↓
**Adjei Boakye et al. (2022)**	2500 AYAs/2500 older cancer survivors	K6<br>Low: 0-5<br>Moderate: 5-13<br>Severe: 13-24<br><br>For moderate distress:<br>**aRR, 1.45 (1.13-1.85)**<br><br>For severe distress:<br><br>**aRR, 1.38 (0.96, 1.99)**	-	-	Work for pay and cancer type (grouped as breast, genitourinary, gynecological, melanoma/skin, thyroid, other cancers) were adjusted for in addition to cohort group	Female – moderate distress ↑<br>Unmarried, uninsured, visiting a mental health professional - severe distress ↑<br>≥2 comorbid conditions, lower educational attainment – moderate and severe ↑
	1605 AYAs/1605 cancer-free peers	For moderate distress:<br>**aRR, 1.21 (0.97, 1.49)**<br><br>For severe distress:<br><br>**aRR, 1.29 (0.94, 1.75)**				
**Hovén et al. (2020)**	2822/28220 cancer-free peers		-	Swedish patient registry<br>ICD-10<br>Mood disorders F3	Calendar period of diagnosis, parents' country of birth, parents' education level (highest) at index, parents'	**-**

				Females 0-5 years since diagnosis:<br><br>**HR, 1.31 (1.09-1.58)**<br><br>Females >5 years since diagnosis:<br><br>**HR, 1.41 (1.17-1.74)**<br><br>Males 0-5 years since diagnosis:<br><br>**HR, 1.32 (1.01-1.72)**<br><br>Males >5 years since diagnosis<br><br>**HR, 1.31 (1.01-1.69)**	marital status at index and parental history of psychiatric disorders.
**Tsuchiya et al. (2020)**	35/196 older cancer survivors	Distress before disclosing cancer status to their friends (5-point scale on a single question)<br><br>**OR, 4.24 (1.67, 10.75)**	-	-	-

Note: ↑ refers to higher risk; HR, hazard ratio; OR, odds ratio; AOR, adjusted odds ratio; RR, risk ratio; [a] Both siblings of childhood cancer survivors and AYA survivors as a comparison group; [b]These were retained covariates after backwards elimination techniques; other variables considered in the model: marital status, personal income, education level, employment status; [c] Based only on questionnaires; [d] Based on the clinical diagnosis (CIDI); [e] 98% (CI); K6, The Kessler Psychological Distress Scale – 6; DIA-X/M-CIDI, Expert System for Diagnosing Mental Disorders; DASS-21, Depression Anxiety and Stress-21; BSI -18, Brief Symptom Inventory – 18; GAD-7, Generalized Anxiety Disorder Scale; PSSCAN-R, The Psychosocial Screen for Cancer; MDD, Major depressive disorder

eTable 7. Main findings on trajectories of psychological distress, anxiety and depression among AYA cancer survivors (n=10)

Study (Year)	Number of AYA cancer survivors	Time of assessment	Assessment tool and cut-off	Prevalence (%)	Differences in rates over time	Findings on predictors
**Outcome: Psychological distress**						
**Chan et al. (2018)**	65 (at baseline)	First medical visit	DT ≥ 4	43.1	Between first medical visit and 1 month (p=0.768)<br>Between diagnosis time and 6 months (p=0.002)<br>Between 1 month and 6 months (p=0.004)	Individuals younger than 24 years old ↑ psychological distress
		1 month after diagnosis		47.7		
		6 months after diagnosis		27.7		
**Chen et al. (2020)**	179 (at 6 months since diagnosis)	6 months since diagnosis	BSI-18≥57	53.2[a]	No tests performed	Being out of work or school, higher scores in neuroticism - distress ↑
		12 months since diagnosis		59.1		
		24 months since diagnosis		52.9		
**Geue et al. (2019)**	514 (at baseline)	Within 4 years of diagnosis (at study start)	HADS ≥ 8 for both anxiety and depression	14.0	No tests performed	-
		After 12 months from study start		15.2		
**Kwak et al. (2013)**	215 (at baseline)	4 months since diagnosis	BSI-18 T ≥63 in two subscales or GSI T ≥63	28.0	No significant changes over time (p>0.05)	Being on treatment and being out of school/work – Psychological distress ↑
		6 months since diagnosis		16.0		
		12 months since diagnosis		23.0		
**Roper et al. (2013)**	40 (at baseline)	After treatment completion	HADS ≥11 for both anxiety and depression	12.5	Significant differences from baseline to 1- and 6-months (p <0.006)	-
		1 month from treatment completion		-		

				-		
		3 months from treatment completion		7.9		
		6 months from treatment completion				
**Tan et al. (2020)**	91 (at baseline)	Median (IQR)			No tests performed	-
		0.8 (0-4.5) months from diagnosis at baseline	DT ≥ 5	45.1		
		1 month after baseline		33.8		
		6 months after baseline		31.4		
		12 months after baseline		25.9		
**Zebrack et al. (2014)**	152	Within 4, 6 and 12 months after diagnosis	BSI-18 T ≥63 in two subscales or GSI T ≥63	27.0 (at 12 months)	Chronic **12%**<br>Delayed **15%**<br>Recovery **20%**<br>Resilient **53%**	-
**Outcome: Anxiety**						
**Ander et al. (2016)**	61 (at baseline)	4-8 weeks after diagnosis	HADS ≥ 9 (13-17 year-old survivors)	15.0	Anxiety - ↓ at 4 years from diagnosis and then increase ↑ up to 10 years from diagnosis (p <0.001)	Female –Anxiety ↑
		10 years after diagnosis	HADS ≥ 8 (older survivors)	29.0		

**Geue et al. (2019)**	514 (at baseline)	Within 4 years of diagnosis (at study start)	HADS ≥ 8	42.2	No tests performed	-
		After 12 months from study start		45.3		
**Jörngården et al. (2007)**	56	Different timepoints:<br>At diagnosis<br>6, 12 and 18 months after	HADS	-	From diagnosis to 18 months ↓ Anxiety ($p < .045$)	-
**Kwak et al. (2013)**	215 (at baseline)	Different timepoints:<br>4, 6 and 12 months after diagnosis	BSI-18<br>T ≥63 in two subscales or GSI T ≥63	-	Anxiety – 4 months vs 6 months ↓ (p=001);<br>6 months vs 12 months ↑ ($p =0.040$);<br>time at diagnosis vs 12 months ($p=0.149$)	-
**Roper et al. (2013)**	40 (at baseline)	After treatment completion	HADS ≥11	13.0	No significant differences	-
		1 month after treatment completion		3.0		
		3 months after treatment completion		5.0		
		6 months after treatment completion		8.0		
**Outcome: Depression**						
**Ander et al. (2016)**	61 (at baseline)	4-8 weeks after diagnosis	HADS ≥ 7 (13-17 year-old survivors)<br>HADS ≥ 8 (older survivors)	18.0	Significant decreases in depression ($p<0.05$)	-
		10 years after diagnosis		0.0		
**Carter et al. (2010)**	71	Pre-operatively	CES-D ≥ 16	49.3	No test performed	-
		3 months after surgery		32.6		
		6 months after surgery		38.6		

		12 months after surgery		19.1		
		18 months after surgery		23.7		
		24 months after surgery		27.5		
**Geue et al. (2019)**	514 (at baseline)	Within 4 years of diagnosis (at study start)	HADS ≥ 8	16.9	-	-
		After 12 months from study start		17.7		
**Jörngården et al. (2007)**	56	Different timepoints:<br>At diagnosis<br>6, 12 and 18 months after	HADS	-	From diagnosis to 18 months ↓ Depression<br>(p ≤0.001)	-
**Kwak et al. (2013)**	215 (at baseline)	Different timepoints:<br>4, 6 and 12 months after diagnosis	BSI-18<br>T ≥63 in two subscales or GSI T ≥63	-	Depression - After 6 months from diagnosis ↓ (p=0.003); no changes afterwards (p=0.210)	-
**Roper et al. (2013)**	40 (at baseline)	At treatment completion, 1, 3 and 6 months after treatment completion	HADS ≥11	-	Depression (p=0.01) significant ↓ from baseline to one month	-

Note: ↑ Represents higher rates of the outcomes in cancer survivors based on the predictor/risk factor and ↓ represents lower rates; When there is no prevalence presented but only screening instrument – prevalence was not reported but only comparisons in means/medians across timepoints; [a]Estimated for all those experiencing distress; BSI -18, Brief Symptom Inventory – 18; HADS, The Hospital Anxiety and Depression Scale; CES-D, Center for Epidemiologic Studies Depression Scale
